# Microbiota-derived acetate protects against respiratory syncytial virus infection through a GPR43-type 1 interferon response

**DOI:** 10.1038/s41467-019-11152-6

**Published:** 2019-07-22

**Authors:** Krist Helen Antunes, José Luís Fachi, Rosemeire de Paula, Emanuelle Fraga da Silva, Laís Passariello Pral, Adara Áurea dos Santos, Greicy Brisa Malaquias Dias, José Eduardo Vargas, Renato Puga, Fabiana Quoos Mayer, Fábio Maito, Carlos R. Zárate-Bladés, Nadim J. Ajami, Marcella Ramos Sant’Ana, Thamiris Candreva, Hosana Gomes Rodrigues, Marcio Schmiele, Maria Teresa Pedrosa Silva Clerici, José Luiz Proença-Modena, Angélica Thomas Vieira, Charles R. Mackay, Daniel Mansur, Mauricio T. Caballero, Jacqui Marzec, Jianying Li, Xuting Wang, Douglas Bell, Fernando P. Polack, Steven R. Kleeberger, Renato T. Stein, Marco Aurélio Ramirez Vinolo, Ana Paula Duarte de Souza

**Affiliations:** 1 Laboratory of Clinical and Experimental Immunology, Infant Center, School of Medicine, Pontifical Catholic University of Rio Grande do Sul, Porto Alegre, 90610-000 RS Brazil; 2 Laboratory of Immunoinflammation, Department of Genetics, Evolution, Microbiology and Immunology - Institute of Biology, University of Campinas, Campinas, 13083007 São Paulo Brazil; 3 Laboratory of Imunobiology, Departament of Microbiology, Immunology and Parasitology, Centro de Ciências Biológicas, Universidade Federal de Santa Catarina, Santa Catarina, 88040900 Brazil; 4 Biological Science Institute (ICB), Passo Fundo University, Passo Fundo, 99052900 State of Rio Grande do Sul Brazil; 5 Clinical Research Center, Hospital Israelita Albert Einstein HIAE, São Paulo, 05652900 Brazil; 6 Molecular Biology Laboratory, Veterinary Research Institute Desidério Finamor, Agricultural Diagnosis and Research Department, Secretariat of Agriculture, Livestock and Irrigation, Eldorado do Sul, 92990000 RS Brazil; 7 Laboratory of Pathology, Healthy Science School, Pontifical Catholic University of Rio Grande do Sul, Porto Alegre, 90610-000 RS Brazil; 8 Laboratory of Iimmunoregulation, Department of Microbiology, Immunology and Parasitology, Federal University of Santa Catarina, UFSC, Florianopolis, 8804900 SC Brazil; 9 Alkek Center for Metagenomics and Microbiome Research, Department of Molecular Virology and Microbiology, Baylor College of Medicine, Houston, TX 77030 USA; 10 Laboratory of Nutritional Genomics, School of Applied Sciences, University of Campinas, Limeira, 13484350 São Paulo Brazil; 11 Laboratory of Nutrients and Tissue Repair, School of Applied Sciences, University of Campinas, Limeira, 13484350 São Paulo Brazil; 12 Institute of Science and Technology, Federal University of Jequitinhonha and Mucuri Valleys (UFVJM), Teófilo Otoni, 39803371 MG Brazil; 13 Department of Food Technology, School of Food Engineering, University of Campinas (UNICAMP) – Cidade Universitária Zeferino Vaz, Monteiro Lobato, 80, Campinas, 13083970 São Paulo Brazil; 14 Emerging viruses study Laboratory, Department of Genetics, Evolution, Microbiology and Immunology, Institute of Biology, University of Campinas, Campinas, 13083970 Brazil; 15 Department of Biochemistry and Immunology, Federal University of Minas Gerais, Belo Horizonte, 31270901 MG Brazil; 16 Biodiscovery Research Institute, Monash University, Clayton, 3800 Australia; 17 Fundación INFANT, Buenos Aires, 1406 Argentina; 18 National Institute of Environmental Health Sciences (NIEHS), NIH, Research Triangle, Durham, 27709 NC USA; 19 Vanderbilt University, EUA, Nashville, 37240 TN USA; 20 Infant Center, School of Medicine, Department of Pediatrics, São Lucas Hospital PUCRS, Porto Alegre, 90610-000 RS Brazil; 21 School of Heath Science, PUCRS, Porto Alegre, 90610-000 RS Brazil

**Keywords:** Immunology, Innate immunity, Bacteria, Virology

## Abstract

Severe respiratory syncytial virus (RSV) infection is a major cause of morbidity and mortality in infants <2 years-old. Here we describe that high-fiber diet protects mice from RSV infection. This effect was dependent on intestinal microbiota and production of acetate. Oral administration of acetate mediated interferon-β (IFN-β) response by increasing expression of interferon-stimulated genes in the lung. These effects were associated with reduction of viral load and pulmonary inflammation in RSV-infected mice. Type 1 IFN signaling via the IFN-1 receptor (IFNAR) was essential for acetate antiviral activity in pulmonary epithelial cell lines and for the acetate protective effect in RSV-infected mice. Activation of Gpr43 in pulmonary epithelial cells reduced virus-induced cytotoxicity and promoted antiviral effects through IFN-β response. The effect of acetate on RSV infection was abolished in *Gpr43*^−^^/^^−^ mice. Our findings reveal antiviral effects of acetate involving IFN-β in lung epithelial cells and engagement of GPR43 and IFNAR.

## Abstract (web summary)

Dietary fibers and SCFAs can exert a protective effect against respiratory syncytial virus (RSV). Here, the authors report that microbiota-derived acetate protects mice against RSV infection via GPR43- mediated type 1 interferon response induction in the lungs.

## Introduction

Respiratory syncytial virus (RSV) is a seasonal pathogen responsible for most cases of severe viral bronchiolitis in children under 2 years of age^1^, and has been estimated to cause up to 118,000 a year deaths worldwide^2^. In addition, there is evidence that suggests severe RSV infection in childhood may be linked with recurrent wheezing and asthma^3,4^. Despite inducing both antibody and T-cell responses following a primary infection, RSV reinfection occurs, even in the absence of detectable antigenic change in virus surface glycoproteins^5^. RSV involves several strategies through evolution to evade host immunity, including modulation of the antiviral type 1 interferon pathway, which is inhibited by RSV NS-1 and NS-2 proteins^6,7^. The development of novel, safe, low-cost therapies to reduce the burden of RSV early in life is highly desirable because there are no clinically approved vaccines.

Several studies demonstrated the relevance of intestinal microbiota in inflammatory and infectious conditions including asthma, colitis, bacterial, and viral infections^8–10^. Bacterial metabolites called short-chain fatty acids (SCFAs), specifically acetate, propionate, and butyrate, are now a well-recognized link between the gut microbiota and host cells. These molecules are generated through metabolism of soluble fibers by components of the intestinal microbiota, such as *Faecalibacterium prausnitzii*, *Bifidobacterium*, *Bacteroides*, and *Eubacterium*^11,12^. The SCFAs have intestinal and systemic effects. They modulate the activation and function of immune cells^8,13–15^ through mechanisms including activation of G-protein coupled receptors (i.e., Gpr41, Gpr43, or Gpr109a) and inhibition of histone deacetylases, and they have been shown to promote gut homeostasis and oral tolerance^16,17^.

Previous investigations suggest that alterations in microbiota composition and in SCFAs intestinal concentrations are also relevant for RSV infection. One previous study associated increased intake of fruits and vegetables (sources of dietary fibers that are used for SCFA production) by pregnant women with newborn protection against severe RSV outcomes^18^. A protective effect of dietary fibers against severe RSV infection is also supported by experimental studies. Mice that received a diet containing oligosaccharides developed a Th1 protective immune response against RSV^19^. Additionally, other studies demonstrated that dietary fibers and SCFAs have a protective effect in the outcome of allergic and inflammatory diseases and in experimental models of respiratory infection^8,15,20–23^.

In the present study we investigate the mechanisms associated with the protective role of a high soluble fiber diet and SCFA production on RSV infection. Our findings indicate an antiviral role for microbiota-derived acetate. This bacterial metabolite, via Gpr43 activation, modulated type 1 response in lung epithelial cells thus protecting against RSV infection.

## Results

### HF diet protects against RSV disease by reducing viral load

**Fig. 1:**
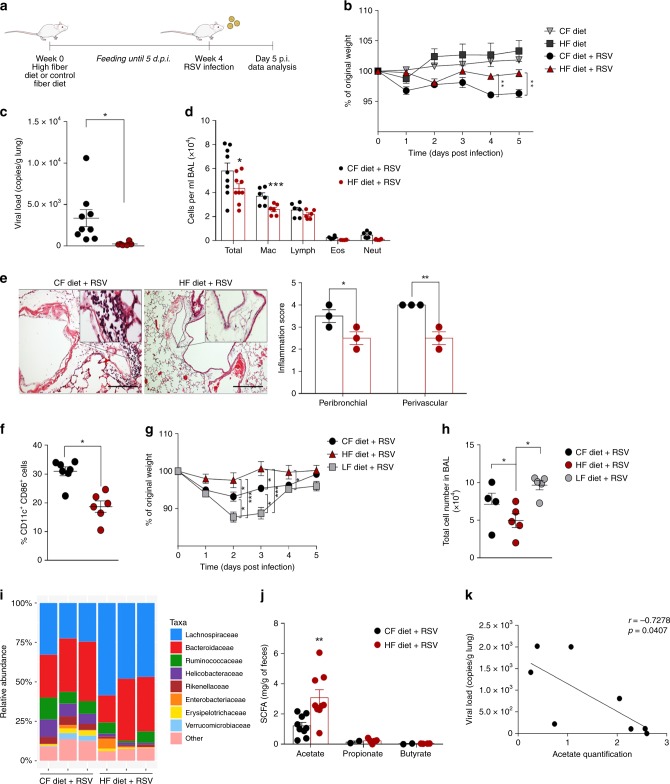
High-fiber diet protects mice against RSV-induced disease. **a** Female BALB/c mice were fed with control fiber (CF) or high-fiber (HF) diets for four weeks before and during RSV infection. Analyses were performed on day five post infection. **b** Percentage of weight loss post infection relative to initial body weight (day 0) (*n* = 9). **c** RSV viral load detected in lung tissue by real-time PCR (viral copies/g of lung tissue) (*n* = 9). **d** Total cell number and differential cell counts in bronchoalveolar lavage fluid (BALF) (*n* = 9). **e** Representative images of lung tissue section stained with hematoxylin and eosin (H&E) and its respective inflammation scores (*n* = 3). Scale bars = 100 μm. **f** Percentage of CD11c^+^CD86^+^ cells in axillary lymph nodes (*n* = 6). **g**, **h** Female BALB/c mice were fed with control fiber (CF), high-fiber (HF) or low-fiber (LF) diet for four weeks before and during RSV infection. Analyses were performed at day five post infection. **g** Percentage of weight loss post infection relative to initial body weight (day 0). **h** Total cell number in BALF (*n* = 5). **i** Analysis of the fecal microbiota composition from mice fed with CF and HF diets at the family level (relative abundance). **j** SCFA quantification in colonic luminal content (mg/g feces) (*n* = 8). **k** Spearman linear correlation between viral load and acetate quantification of animals fed with control and high-fiber diet. Data in **a**–**e** and **j** are from two independent experiments. All data are expressed as mean ± SEM and were compared using Kruskal–Wallis. In the other cases, Mann–Whitney was used. *p < 0.05, **p < 0.01, ***p < 0.001. Data in **b**, **c**, **d**, **g**, **h**, **j** are provided as a Source Data file

Mice were fed a control diet [CF; with cellulose (AIN-93M, American Society for Nutrition, USA)], or a high-fiber diet (HF), which contained cellulose and pectin from citrus^24^for 4 weeks and then infected with RSV intranasally (Fig. 1a). HF diet protected mice from RSV-induced weight loss (Fig. 1b) and also reduced lung viral load (Fig. 1c). HF diet significantly reduced the numbers of total cells in the bronchoalveolar lavage fluid (BALF), mainly attributed to reduction of macrophages (Fig. 1d). HF diet also reduced lung histological inflammatory score (Fig. 1e) and CD11c^+^CD86^+^ cells in the axillary lymph nodes (Fig.1f). No significant effect of the diet was found on CD8^+^CD69^+^CD62L^−^or CD4^+^CD25^+^FoxP3^+^ cells in the lymph nodes (Supplementary Fig. 1, A). HF diet also did not change the pattern of cells present in the lung (Supplementary Fig. 1, B). To confirm the importance of fiber in protection against RSV infection, the same experiment was performed with mice on a low-fiber diet (cellulose was replaced by starch). Mice fed a low-fiber diet developed a worse RSV infection with accentuated loss of body weight and increased numbers of inflammatory cells in BALF after infection when compared to mice on the CF or HF diets (Fig. 1g and h).

HF diet affected the composition of the gut microbiota in RSV-infected mice (Supplementary Fig. 2, A–C). Although there was no statistical difference at the phylum level or in bacterial diversity, we found an increase in the relative abundance of *Lachnospiraceae spp*. (phylum Firmicutes, class Clostridia) (Fig. 1i and Supplementary Table 1). The Lachnospiraceae family has been associated with SCFA production^25^. Indeed, we found increased acetate concentrations in mice fed with the HF diet (Fig. 1j) and this correlated negatively with viral loads in the lung (Fig. 1k). These findings demonstrate inhibition of RSV-induced disease by HF diet intervention and suggest a role for acetate in this protection.

### Microbiota is essential for protection against RSV infection

**Fig. 2:**
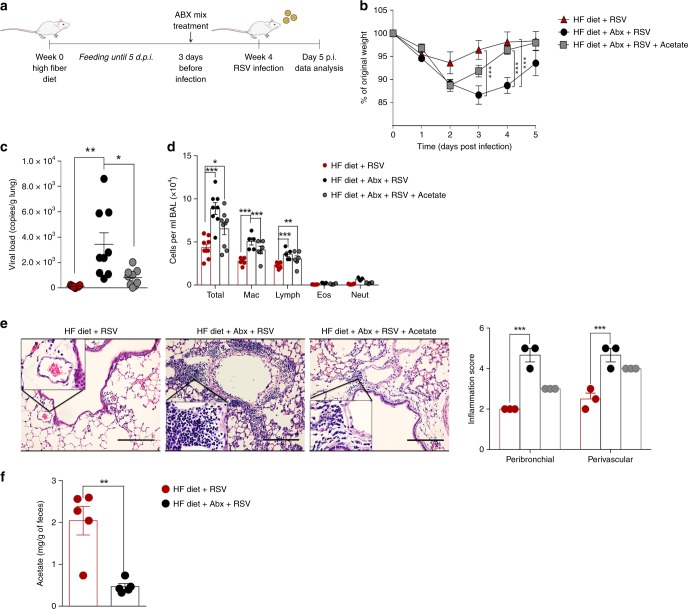
Microbiota and acetate are essential to HF diet protection against RSV infection. **a** BALB/c mice were fed a HF diet for four weeks before and during RSV infection. Three days before infection mice received an antibiotic mix (Abx) in the drinking water. Acetate was administered in drinking water after removal of abx treatment. Analyses were performed on day five post infection. **b** Percentage of body weight loss post infection relative to initial weight (day 0) (*n* = 9). **c** RSV viral load detected in lung tissue by real-time PCR (viral copies/g lung tissue) (*n* = 9). **d** Total cell number and differential cell counts in BALF (*n* = 9). **e** Representative images of lung tissue section stained with H&E and its respective inflammation scores (*n* = 3). Scale bars = 100 μm. **f** Acetate quantification in colonic luminal content (mg/g feces) (*n* = 5). Data are from two independent experiments. All data are expressed as mean ± SEM. The groups were compared using Kruskal–Wallis, except in Fig. 2f, in which the comparison between groups was done using Mann–Whitney test. *p < 0.05, **p < 0.01, ***p < 0.001. Data in **b**, **c**, **d**, **f** are provided as a Source Data file

We next explored whether or not microbiota and the SCFA acetate are involved in protection against RSV infection. HF-diet-fed mice were given an antibiotic cocktail in drinking water 3 days before RSV inoculation (Fig. 2a) and disruption of the gut microbiota by antibiotic treatment was confirmed by a significant decrease in bacterial load in feces (Supplementary Fig. 2, D). The protection against weight loss mediated by the HF diet was abolished when mice were treated with antibiotics (Fig. 2b). Similarly, reduction in lung viral load was not found when mice were treated with antibiotics (Fig. 2c) and we found increased total number of cells in BALF, including macrophages and lymphocytes (Fig. 2d). Antibiotic treatment also increased inflammatory cell infiltration in the lung (Fig. 2e). A 4-fold reduction of acetate concentrations was found in the colonic content 5 days after antibiotic cessation (Fig. 2f). Propionate and butyrate concentrations were also reduced in antibiotic treated mice to a point below the limit of detection (Supplementary Fig. 2E). Acetate supplementation of antibiotic-treated mice reduced body weight loss caused by RSV infection (Fig. 2b) and partially recovered the phenotypes previously observed [e.g., reduction in lung viral load (Fig. 2c) and inflammatory cell infiltration of the lung (Fig. 2d and e)]. Although we cannot rule out the involvement of other mechanisms in the protection observed after HF diet, the data from these experiments support the hypothesis that HF diet protection against RSV infection is dependent on a preserved intestinal microbiota, and acetate is an important mediator of these effects.

### Acetate treatment protects mice against RSV infection

**Fig. 3:**
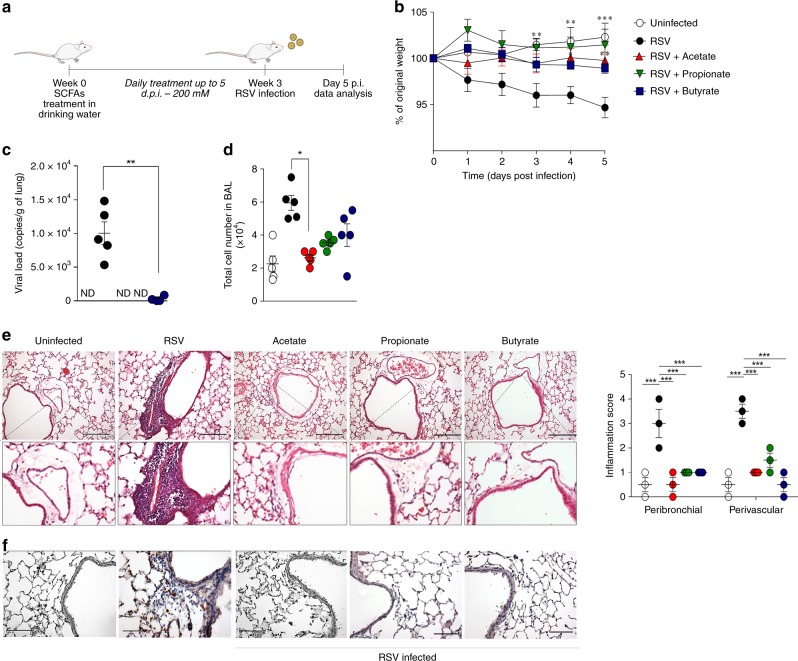
SCFAs pre-treatment protects mice against RSV infection. **a** BALB/c mice were given, sodium acetate, sodium propionate or sodium butyrate in sterile drinking water at a final concentration of 200 mM for 3 weeks before and during RSV infection. Analyses were performed on day five post infection. **b** Percentage body weight loss post infection relative to initial weight (day 0) (*n* = 5). **c** RSV viral load detected in lung tissue by real-time PCR (*n* = 5). **d** Total cell number in BALF (*n* = 5). E, Representative hematoxylin and eosin (H&E)-stained lung tissue images and its respective inflammation score. Scale bars = 100 μm. **f** Immunohistochemical (IHC) staining using an anti-RSV Fusion Protein antibody. Scale bars 200 μm. The results are expressed as mean ± SEM. ND not detected. Statistical significance between the groups was determined by Kruskal–Wallis, except in **c**, in which Mann–Whitney was used to compare the groups. *p < 0.05, **p < 0.01, ***p < 0.001. Data in **b**–**d** are provided as a Source Data file

**Fig. 4:**
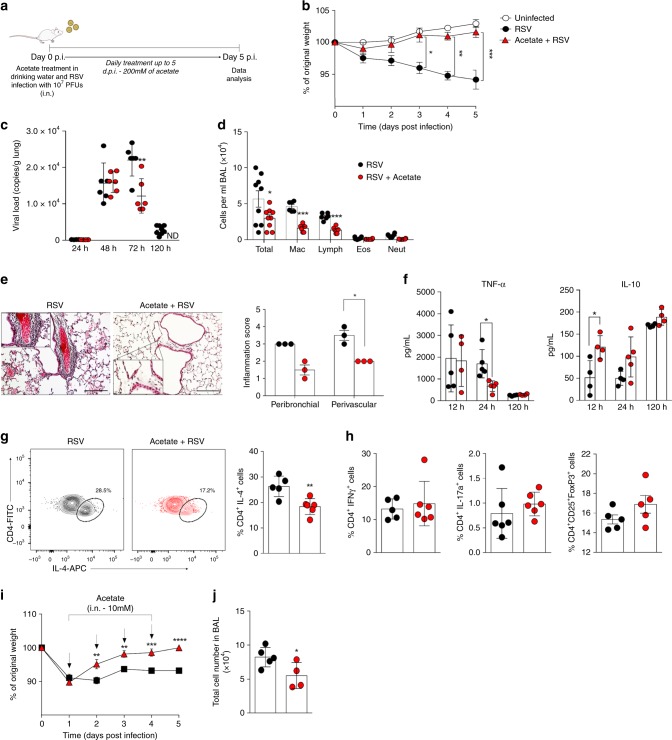
Acetate treatment protects mice against RSV infection. **a** BALB/c mice were simultaneously infected with RSV and treated with 200 mM acetate in the drinking water. Analyses were performed on day five post infection. **b** Percentage body weight loss post infection relative to initial weight (day 0) (*n* = 9). **c** RSV viral load was measured 24, 48, 72, and 120 h after infection detected in lung by real-time PCR (viral copies/g lung tissue) (24, 48, and 72 h, *n* = 5; 120 h, *n* = 9). ND = not detected. **d** Total cell number and differential cell counts in BALF (*n* = 9). **e** Representative images of lung tissue stained with H&E and its respective inflammation scores (*n* = 3). Scale bars = 100 μm. **f** Release of TNF-α and IL-10 in BALF measured at different times after infection (*n* = 4). **g** Percentage CD4 + IL-4 + T cells in lung and its representative FACS profile (*n* = 6). **h** Percentage of IFNγ ^+^ , IL-17a ^+^ and FoxP3  CD4 T cells in the lung. The representative FACS profiles are shown in Supplementary Fig.3 A and B. **i**, **j** Female BALB/c mice were infected with RSV (10^7^ PFU/ml) and 24 h after infection started the treatment with acetate through the intranasal route (10 mM). Treatment was performed daily up to 5 days after infection. **i** Percentage body weight loss post infection relative to initial weight (day 0) (*n* = 5). **j** Total cell number in BALF (*n* = 5). All data are expressed as mean ± SEM. Data in **b**, **c**, **d** are from two independent experiments. Statistical significance between the groups was determined by Kruskal–Wallis, except in **g**, **h**, **j**, in which Mann–Whitney was used to compare the groups. *p < 0.05, **p < 0.01, ***p < 0.001. Data in **b**, **c**, **d**, **g**, **i**, **j** are provided as a Source Data file

Acetate was added to the drinking water before infection, in a manner similar to the experiments performed with diet (Fig. 3a). The treatment caused lower weight loss after RSV infection (Fig. 3b), an undetectable lung viral load (Fig. 3c), a decrease in total cell numbers in the BALF (Fig. 3d) and reduced inflammatory cells in the lungs (Fig. 3e). RSV-F protein was not detected by immunohistochemical analysis in lung tissue of mice pre-treated with acetate, confirming the protective effect of acetate against virus infection (Fig. 3f). Propionate and butyrate pre-treatment caused similar protective effects (Fig. 3a–f). These findings indicate a prophylactic effect of SCFAs on RSV infection. We also treated mice with acetate in the drinking water simultaneously with intranasal RSV instillation, in order to test for a possible therapeutic effect of acetate (Fig. 4a). Notably, acetate protected mice against weight loss caused by infection (Fig. 4b). Acetate started to reduce the viral load significantly at day 3 post infection, culminating in an undetectable viral load in the lung on day 5 (Fig. 4c). The treatment reduced cell numbers in BALF, including a significant reduction in macrophage and lymphocyte amounts (Fig. 4d). Hence, acetate treatment reduced the inflammatory score in the lung (Fig. 4e), and reduced TNF-α and increased IL-10 concentrations in BALF (Fig. 4f). Acetate treatment also reduced IL-4-producing CD4 T cells in the lung and axillary lymph nodes, but the same reduction was not observed for IFN-γ-, IL-17a-producing-T cells or FoxP3-positive cells (Fig. 4g, h and Supplementary Fig. 3, C). No significant differences were found for CD11c^+^CD86^+,^ and CD8^+^CD69^+^CD62L^−^in the axillary lymph node and in the lung (Supplementary Fig. 3, D and E). We also found that acetate treatment administered by intranasal route (24 h after infection) protected against weight loss and cell recruitment to lung caused by infection (Fig. 4i and j).

### Acetate induces a IFN-1 response in pulmonary cells

**Fig. 5:**
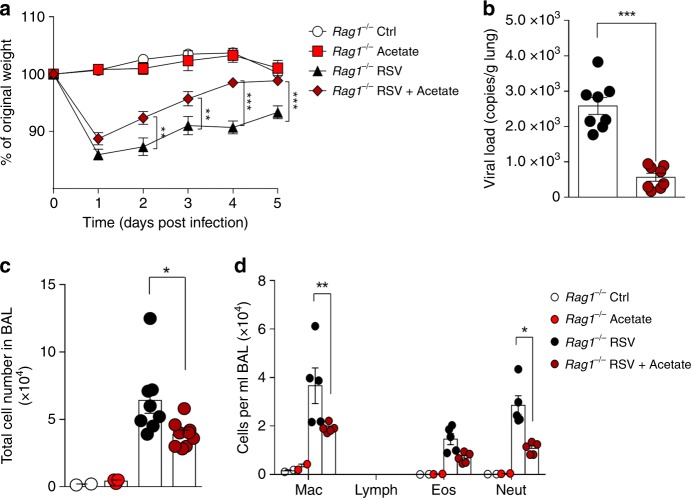
Acetate treatment protects against RSV infection in the absence of T cells. **a**–**d** Female *Rag1* knockout mice (background C57BL/6) were simultaneously infected with RSV and treated with 200 mM acetate in drinking water. Analyses were performed on day five post infection. **a** Percentage body weight loss post infection relative to initial weight (day 0) (*n* = 8). Ctrl = untreated and uninfected mice. **b** RSV viral load detected in lung tissue by real-time PCR (viral copies/g lung tissue) (*n* = 8). **c** Total cell number and **d** differential cell counts in BALF (**c**, *n* = 8; **d**, *n* = 5). All data are expressed as mean ± SEM. Data are from two independent. Statistical significance between the groups was determined using Kruskal–Wallis test except in **b**, in which Mann–Whitney was used. *p < 0.05, **p < 0.01, ***p < 0.001. Data in **a**–**d** are provided as a Source Data file

Based on our findings that the HF diet and acetate treatment reduced IL-4-producing CD4 T cells, we tested whether or not T cells mediated the protective effect of acetate against RSV-induced disease. *Rag*1^−/−^ mice were treated with acetate using the same protocol described in Fig.4 a. Acetate treatment protected against RSV-induced body weight loss (Fig. 5a), reduced lung viral load even in the absence of B/T-lymphocytes (Fig. 5b) and decreased the number of macrophages, neutrophils, and the total number of cells in the BALF (Fig. 5c and d). These data indicate that acetate has a B/T-cell-independent protective effect against RSV infection.

**Fig. 6:**
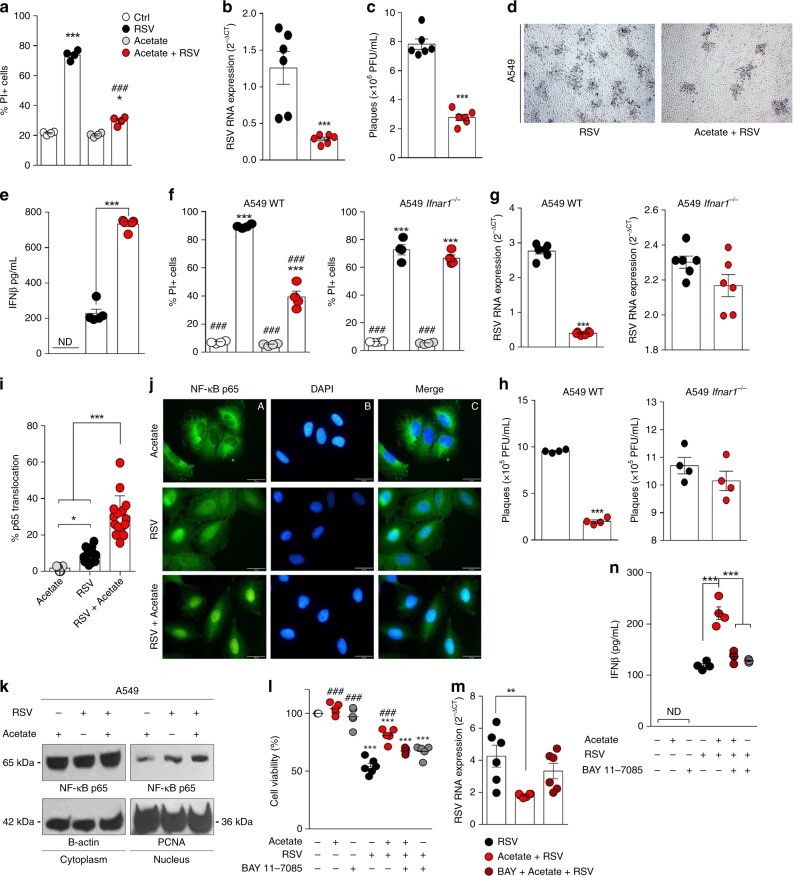
Acetate treatment mediated IFN-β response in pulmonary cells lines reducing viral load. **a**–**d** A549 cells were pre-treated with 260 µM acetate for 24 h and infected with RSV for 96 h. **a** Percent of PI (propidium iodide) positive cells detected by flow cytometry. **b** RSV RNA levels detected using real-time PCR (2^-ΔCt^). **c** Quantification of RSV PFUs. **d** Representative images of viral titration assay indicating viral plaques. Lysis plate titration was performed using an anti-RSV antibody. **e** IFN-β protein levels in supernatants of A549 cells pre-treated with 260 µM acetate for 24 h and infected with RSV for further 24 h. **f** Cellular viability of A549 WT and A549 *Ifnar1*-/- assessed using PI by flow cytometry. **g** RSV RNA levels detected using real-time PCR (2^-ΔCt^ analysis). **h** Quantification of RSV by PFU assay. **i** Quantification of translocated NF-kB p65 subunit in A549 cells pre-treated with 260 µM acetate 24 h and infected for 2 h (*n* = 2 experiments in sextuplicates). **j** Fluorescence images of NF-kB p65 (green—**A**) and cell nuclei using DAPI (blue—**B**). Panel C shows data co-localization (merge). Scale bars = 20 µm. **k** Western blot analysis of NF-kB p65 in cytoplasmic and nuclear lysates. Cytoplasmic protein bands were normalized by β-actin and nuclear protein bands were normalized by PCNA. L-N, A549 cells were pre-treated 1 h with 2 µM BAY 11–7085. Cells were then treated with 260 µM acetate for 24 h and infected. **l** Cell viability was evaluated by MTT assay. **m** RSV RNA levels detected using real-time PCR. **n** IFN-β protein levels detected by ELISA in the culture supernatant 24 h after infection. All data are expressed as mean ± SEM. Data in **a**, **f**, **h**, **m** are quadruplicate mean from 3 experiments. Data in **b**, **g**, **l**, **m** are sextuplicate mean from two experiments. * Significant difference relative to ctrl; # significant difference relative to RSV. Statistical significance was determined with Kruskal–Wallis, except in **b**, **c**, **g**, **h**, in which Mann–Whitney. *p < 0.05, **p < 0.01, ***p < 0.001. Data in **a**, **b**, **c**, **e**, **f**, **g**, **h**, **i**, **k**, **m** are provided as a Source Data file

We hypothesized that acetate has a direct antiviral effect on pulmonary cells, given its significant effect on diminishing viral loads in the lung. Human pulmonary epithelial cells (A549 and MRC-5) were treated with acetate at different concentrations and time periods (6 h, 12 h (Supplementary Fig. 4, A), or 24 h before infection (Supplementary Fig. 4, A, B) or 2 h after infection with RSV (Supplementary Fig. 4C)). Acetate treatment 24 h before infection protected cells from cellular death caused by RSV (Fig. 6a and Supplementary Fig. 4, A, B, and D), and reduced RSV F protein RNA levels and viral plaque formation (Fig. 6b, c and d and Supplementary Fig. 4, E, F and G). Propionate and butyrate also protected epithelial lung cells in vitro (Supplementary Fig. 4, A). We then tested the same treatments on Vero cells. The protective effect against RSV-induced cell death was not observed in this cell line (Supplementary Fig. 5, A–D). Because Vero cells contain a mutation, which impairs the production of IFN-β^26^, we hypothesized that acetate protection could be in part attributed to induction of this cytokine. To test this, we measured IFN-β in the supernatant of MRC-5 and A549 cells and found that cells treated with acetate and infected with RSV presented an increment of IFN-β levels (Fig. 6e and Supplementary Fig 5, E). There was not a significant increase in the interferon lambda gene expression in A549 cells treated with acetate (Supplementary Fig. 5, F). Acetate did not protect CRISPR/Cas9-generated A459 type 1 interferon receptor knockout (*Ifnar*^−/−^) cells from RSV-induced cell death (Fig. 6f), and had no effect on virus replication (Fig. 6g, h and Supplementary Fig 5, G). To better understand the mechanisms by which acetate mediated IFN-β production, we investigated the role of NF-kB in this process. NF-κB has an important role in the signal transduction pathway that recognized viral nucleic acids during RSV infection and can lead to induction of the IFN-β^27,28^. We first investigated whether acetate mediated NF-κB activation and we found that treatment increased the nuclear translocation of p65 in A549 cells infected with RSV (Fig. 6i and j). This finding was corroborated by western blot results in which we observed an increase of p65 in nuclear lysate of acetate-treated and -infected cells (Fig. 6k). Pre-treatment of the cells with a NF-κB pathway inhibitor (BAY 11–7085, an irreversible inhibitor of IκBα phosphorylation) abolished the protective effect (Fig. 6l and m). In addition, the induction of IFN-β in A549 cells was dependent on NF-κB activation (Fig. 6n). These findings indicate that the in vitro protective effect of acetate against RSV infection is mediated by IFN-β and IFNAR engagement, and this mechanism is associated with NF-κB activation.

### Acetate protects against RSV in an IFNAR-dependent manner

**Fig. 7:**
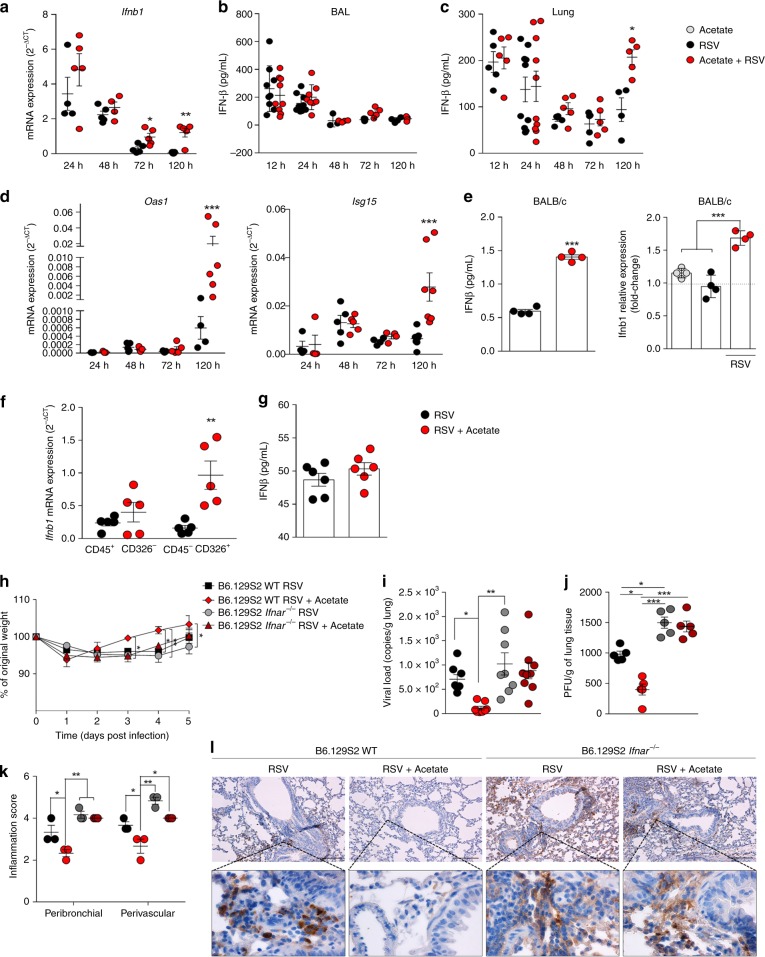
Acetate protects against RSV infection in an IFNAR-dependent manner. Female BALB/c mice were treated with 200 mM acetate in drinking water and infected with RSV (10^7^ PFU/mL) for 24, 48, 72, and 120 h. **a** *Ifnb1* gene expression in the lung detected at different time points after infection (2-ΔCt analysis in real-time PCR) (*n* = 5). **b** IFN-β protein detected in the BALF or lung homogenates **c** at different time points after infection (*n* = 5). **d** *Oas1* and *Isg15* gene expression in the lung (2-ΔCt analysis in real-time PCR) (*n* = 5). **e** Pulmonary epithelial cells from female naïve BALB/c mice were treated with acetate (260 µM) for 24 h and then infected with RSV (10^4^ PFU/mL) for a further 24 h to detect IFN-β production (by ELISA) and *Ifnb1* expression (fold change compared to untreated/uninfected control) (*n* = quadruplicate mean of 4 animals). **f** *Ifnb1* expression of CD45^+^CD326^-^ and CD45^-^CD326^+^ sorted cells from lung of mice acetate-treated and RSV-infected for 72 h (n = 5). **g** IFN-β production by alveolar macrophages of mouse untreated or treated with acetate in drinking water (200 mM) for 5 days and infected ex vivo with RSV (10^4^ PFU/ml) for 24 h (*n* = 6). Gate strategy, pre-sorting and post-sorting panel are shown in Supplementary Fig. 9. **h**–**l** Wild-type and *Ifnar*-/- mice were simultaneously infected with RSV and treated with 200 mM acetate in drinking water. Analyses were performed on day 5 post infection. **h** Percentage body weight loss post infection relative to initial weight (day 0) (*n* = 8). **i** RSV viral load detected in lung tissue by real-time PCR (viral copies/g lung tissue) (*n* = 8). **j** Viral titer in the lung (PFUs/g lung tissue) (*n* = 5). **k** Inflammation score of lung histology (*n* = 3). **l** Immunohistochemistry staining of RSV in lung tissue sections. All data are expressed as mean ± SEM. Data are from two independent experiments. Statistical significance between groups was determined with Kruskal–Wallis, except in **e**, in which Mann–Whitney was used. *p < 0.05, **p < 0.01, ***p < 0.001. Data in **a**, **b**, **c**, **d**, **f**, **h**, **i**, **j** are provided as a Source Data file

We initially tested the possible role of acetate in IFN-β production and found increased expression of *Ifnb1*, but not *Ifna1*, in the lung of RSV-infected mice treated with acetate compared to untreated RSV-infected mice 3 and 5 days after infection (Fig. 7a and Supplementary Fig 6A). We found increased concentrations of IFN-β in BALF and lung of acetate-treated mice 72 and 120 h after infection (Fig. 7b and c). Acetate treatment also induced the expression of the IFN-1 stimulated genes (ISGs), *Oas1* and *Isg15* (Fig. 7d). To confirm that acetate was inducing IFN-β production in pulmonary epithelial cells, we performed an ex vivo assay. We collected fresh lung epithelial cells from naive mice and cultured them. The cells were pre-treated with acetate for 24 h and then infected with RSV for another 24 h. Acetate treatment increased IFN-β production (measured in the culture supernatants), as well as *Ifnb1* expression compared to non-treated RSV-infected cells (Fig. 7e). In addition, epithelial cells (CD45^-^CD326^+^) and leukocytes (CD45 ^+^ CD326^−^) sorted from dissociated lungs of mice treated with acetate and infected with RSV were analysed for the expression of *Ifnb1*. As demonstrated in Fig. 7f, acetate treatment increased the expression of this gene in epithelial cells, but not in the leukocyte compartment. There was no significant difference between the ISGs and interferon lambda expression (Supplementary Fig. 6B and C). These findings were corroborated by the fact that we did not find any ex vivo or in vitro effect of acetate on IFN-β production by alveolar macrophages (Fig. 7g). HF diet induced IFN-α and IFN-β production (Supplementary Fig. 6D and E). Consistent with our in vitro data, acetate protection against RSV infection was abolished in the absence of type 1 interferon receptor (*Ifnar−/*−) in vivo. No significant differences were found in body weight loss (Fig. 7h), total cell numbers in the BALF (Supplementary Fig 6F), lung viral load, viral titer in the lung (Fig. 7i and j and Supplementary Fig. 6G), or lung inflammatory score (Fig. 7k and Supplementary Fig 6H) between *Ifnar−/−* mice treated with acetate or vehicle. Moreover, in the absence of the receptor, acetate was unable to cause viral clearance in the lungs (Fig. 7l). No changes in DCs, T CD8, and T reg cell populations were found (Supplementary Fig 6I and J). These data confirm that the acetate protection against RSV infection is dependent on the presence of *Ifnar* and it is mediated by IFN-β in pulmonary epithelial cells.

### *IFNAR1*rs2257167 C allele protects for severe RSV disease

To evaluate the importance of *Ifnar* in RSV infection, we queried the NIEHS TagSNP database to identify a tagSNP that provided adequate gene coverage (Supplementary Fig. 7). *IFNAR1* SNP rs2257167 was selected for genotyping DNA samples of children from a case-control study with healthy full-term infants (<1 year of age) presenting with bronchiolitis (*n* = 401)^29^.

Detailed clinical and demographic data have been previously described^29^. The phenotype of interest was clinically defined as “severe” or “mild” RSV disease and served as a dichotomous dependent variable. The estimated minor allele frequency (MAF) for the *IFNAR1* SNP rs2257167 (located on chromosome 21:33343393) is 0.2288 in the 1000 genomes population. In our study population, the overall MAF was 0.2687 and, in the sub-population of RSV-positive patients (*n* = 401), the MAF was 0.2681 (Supplementary Table 2). We hypothesized three potential effects of this functional SNP and tested these with logistic regression models. We found significant associations in all models tested (Supplementary Table 3). In testing for the variant effects, we found the odds ratios were 0.5932 and 0.3549 for the “dominant model” and “recessive model” with the 95% confidence interval at [0.3966, 0.8874] and [0.1694, 0.7437], respectively. Thus, the variant C (Leu168) is protective against severe disease for those individuals infected with RSV. Incorporation of breastfeeding increased genetic effects in all three models (*p*-values were 0.0014, 0.0098, and 0.0054, respectively).

### GPR43 is essential for acetate protection against RSV

**Fig. 8:**
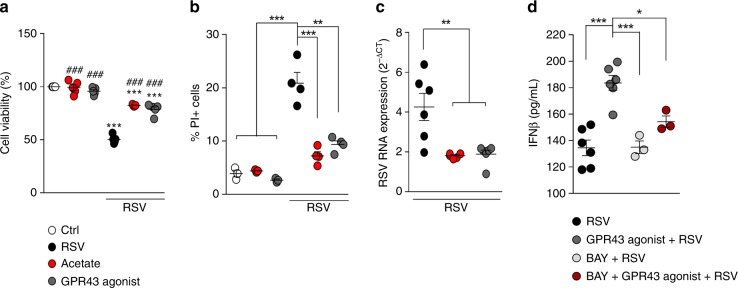
Specific activation of GPR43 protects against in vitro RSV infection. A549 cells were treated with 260 µM acetate or a GPR43-synthetic agonist (10 µM 4-CMTB) for 24 h and then infected with RSV (10^4^ PFU/ml). Four days later response parameters were analyzed. **a** cell viability was accessed by MTT assay. **b** Percent of cell death was determined by PI staining. **c** RSV RNA levels were detected using real-time PCR (2-ΔCt analysis). **d** Concentrations of IFN-β in the cell supernatant 12 h after infection were detected by ELISA. All data are expressed as mean ± SEM. Data in **a** and **b** are quadruplicate mean of two experiments. Data in **c** and **d** are sextuplicate mean of one experiment. Statistical significance was determined with Krukal-wallis. **p < 0.01, ***p < 0.001. Data in **b**–**d** are provided as a Source Data file

Given the importance of G-protein coupled receptors (i.e., GPR41, GPR43, and GPR109a) in SCFA responses^16^, we investigated whether or not the antiviral effect of acetate was mediated by activation of these receptors. The pulmonary epithelial cell lines MRC5 and A549 express *Gpr43*, *Gpr41*, and *Gpr109A* (Supplementary Fig. 8A). Both, GPR41 and GPR43, can be activated by acetate. Considering this, we repeated the in vitro experiments using a potent and selective GPR43-synthetic agonist^30^. The GPR43 agonist caused the same pattern of response observed with acetate. It protected the cells against infection (Fig. 8a and b), reduced the virus load (Fig. 8c) and induced IFN-β (Fig. 8d), but at an earlier time point than acetate (12 h) (Fig. 8d). In accordance with our previous results, the effect of the synthetic agonist on IFN-β production was dependent on NF-kB activation (Fig. 8d). These findings indicate that GPR43 may account for IFN-β production in cells treated with acetate.

**Fig. 9:**
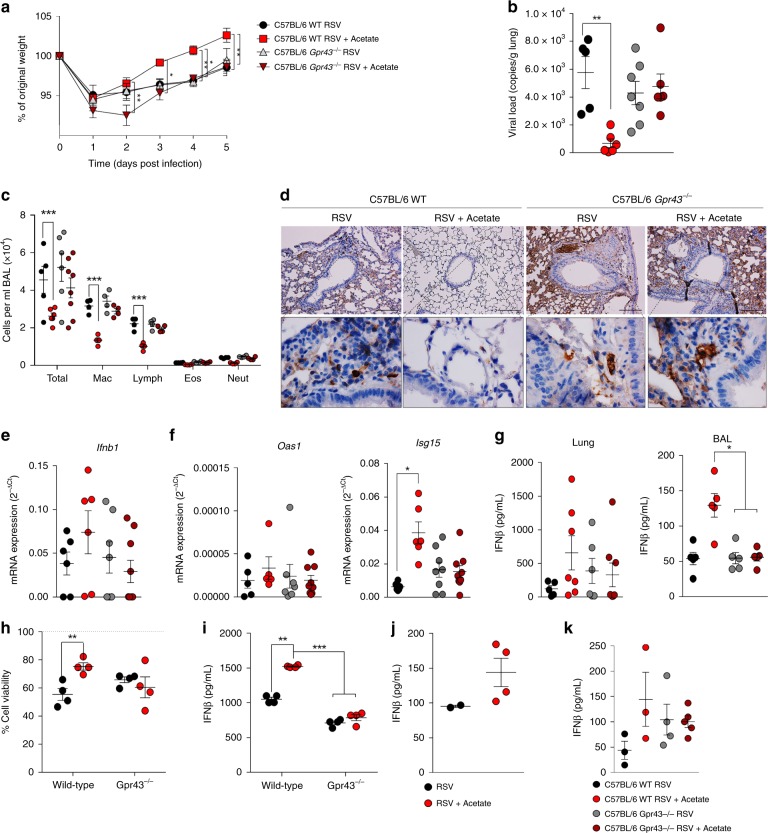
GPR43 is necessary for acetate protection of RSV-induced disease. **a**–**g** Gpr43 (G-protein coupled receptor 43) knockout mice (background in C57BL/6) and their controls (WT) were infected with RSV and treated with 200 mM acetate in drinking water. Analyses were performed on day five post infection. **a** Percentage weight loss post infection relative to original weight (day 0) (WT, *n* = 6; *Gpr43−/−*, *n* = 9). **b** RSV viral load detected in lung tissue by real-time PCR (viral copies/g of lung tissue) (WT, *n* = 6; *Gpr43−/−*, *n* = 9). **c** Total cell number and differential cell counting in BALF(WT, *n* = 6; *Gpr43−/−*, *n* = 8). **d** Immunohistochemistry staining of RSV in lung tissue sections. **e** mRNA expression of Ifnb1 in the lung (2-ΔCt analysis) (WT, *n* = 6; *Gpr43−/−*, *n* = 8). **f** mRNA expression of *Oas1* and *Isg15* genes in the lung (2-ΔCt analysis) (WT, *n* = 6; *Gpr43−/−*, *n* = 8). **g** IFN-β protein levels detected in the BALF or lung homogenate (WT, *n* = 4; *Gpr43−/−*, *n* = 6). **h**, **i** Pulmonary epithelial cells from female C57BL/6 wild-type and *Gpr43−/− *mice were treated with 260 µM acetate for 24 h and then infected with RSV (10^4^ PFU/mL) for 24 h or 4 days. **h** cell viability evaluated by MTT assay. Control untreated/uninfected is showed as the dotted line. **i** IFN-β levels detected in supernatant of primary mouse lung cells culture (*n* = quadruplicate mean of 4 animals). **j**, **k**, Female C57BL6 wild-type and *Gpr43−/−* mice were infected with RSV (10^7^ PFU/ml) for 24 h and then treated with acetate through the intranasal route (10 mM). After 24 h the lung was collected and processed for detection of IFNβ production by ELISA. All data are expressed as mean ± SEM. Data in **a**–**g** are from two independent experiments. Statistical significance between the groups was determined with Kruskal–Wallis. *p < 0.05, **p < 0.01, ***p < 0.001. Data in **a**, **b**, **c**, **f**, **g**, **i** are provided as a Source Data file

To further investigate the role of GPR43 on acetate effects, we repeated the in vivo experiments with *Gpr43*^−/−^ mice using the same protocol described in Fig. 4a. Acetate protection against RSV-induced disease was abolished in the absence of GPR43. There were no differences between the acetate-treated and control groups in *Gpr43*^−/−^ mice with respect to body weight loss (Fig. 9a), viral load in the lung (Fig. 9b) and cellularity in the BALF (Fig. 9c), and these mice also had no protection conferred by acetate against inflammation (Supplementary Fig 8B). The acetate antiviral effect was also abrogated in these mice (Fig. 9d). In addition, acetate did not induce *Ifnb1*, *Oas1*, and *isg15* gene expression (Fig. 9e and f) or increase IFN-β production (Fig. 9g) in the *Gpr43*^−/−^ mice. To verify whether the loss of the protective effect of acetate was due to an effect on epithelial cells, we performed the same ex vivo analysis described above using lung epithelial cells isolated from C57BL/6 wild-type and *Gpr43*^*−/−*^ mice. This showed that the acetate treatment protected the mouse epithelial cells from cell death caused by the virus and this effect was abolished in the absence of GPR43 (Fig. 9h). The induction of IFN-β by acetate was evident when using WT mouse lung epithelial cells but absent when the experiment was performed with *Gpr43−/*− cells (Fig. 9i). This response was also found using the synthetic GPR43 agonist instead of acetate (Supplementary Fig 8C). In line with these results using the intranasal route for acetate administration, we found a 3-fold increase in IFN-β levels in BAL at 24 h after infection (Fig. 9j). The same experiment was repeated in GPR43 deficient mice but no differences between treated and control were found (Fig. 9k). These data indicate that the acetate antiviral activity against RSV, which involves induction of IFN-I production, is also mediated by GPR43 activation.

To better understand the potential genetic contribution of *Ifnar1* and *Gpr43* (*Ffar2*) to the response to RSV, we searched the Jackson Laboratory Mouse Phenome Database for informative SNPs in both the genes to determine whether they associated with RSV-induced phenotypes identified in a previous study of 30 inbred strains of mice^31^. To be considered for analysis, the minor allele SNPs must have been present in ≥10% of the phenotyped inbred strains. A non-synonymous coding SNP rs31418313 (A/G) in *Ifnar1* that causes an arginine to histidine substitution in the 274 amino acid residue was significantly (*P* < 0.05) associated with greater mean numbers of BAL polymorphonuclear leukocytes (PMNs) and % loss of body weight compared to strains of mice with the wild-type A allele (Supplementary Table 4). A non-synonymous coding SNP rs47500117 (T/G) in *Gpr43* that causes an isoleucine to leucine substitution in the 291 amino acid residue significantly (*P* < 0.05) associated with greater BAL total protein (a marker of epithelial permeability) compared to strains of mice with the wild-type T allele (Supplementary Table 4). Finally, we found that another *Gpr43* non-synonymous coding SNP rs236386097 (T/C; methionine to valine, residue 298) that was associated with significantly (*P* < 0.05) greater numbers of BAL total cells, monocytes, and protein as well as intraepithelial cell mucus in strains with the C allele compared to wild-type strains (T allele). These results confirmed the importance of IFNAR and GPR43 during RSV infection and provide rationale for further investigating the potential role of homologous candidate functional SNPs in human populations.

## Discussion

We identified a distinct mechanism by which acetate, a microbiota-derived metabolite, impacts immune responses during viral infection in the lung. Acetate protected against RSV-induced disease by improving type 1 interferon responses and increasing interferon-stimulated gene expression in lung epithelial cells, through a mechanism that involves activation of the membrane receptor GPR43. These findings highlight the relevance of the gut microbiota and associated metabolites in non-intestinal inflammatory and infectious conditions. Our data also support the concept of using of high-fiber diets, fiber-enriched formulas or even acetate, as inexpensive interventions for prevention and treatment of bronchiolitis caused by RSV.

Lynch et al. (2018) demonstrated a role for the intestinal microbiota and propionate, in the early protection against bronchiolitis and the development of asthma in adult mice^21^. In our study, we found a similar prophylactic effect after oral supplementation with propionate, acetate, or butyrate before RSV infection. Interestingly, we also found beneficial effects when treating mice with acetate by oral route simultaneously with infection and by intranasally 24 h after infection. This latter result indicates that acetate may be useful in the treatment of RSV infection. In contrast to Lynch et al. (2018)^21^, who showed that the propionate protection effect was dependent on T reg cells, the acetate effect in our study involved mechanisms that were T-cell-independent. We found that acetate modulated IFN-β response in vitro in human pulmonary cell lines and in vivo in the lungs of RSV-infected mice. This mechanism was relevant for the protection conferred by acetate since we found this effect against RSV infection was abrogated in the absence of type 1 interferon receptor (IFNAR) in vivo and in vitro. Previous studies have already described the function of IFN-β during RSV infection^32–34^. In addition, the importance of the microbiota for the production of IFN-1^35^, and protection against respiratory viral infection had been demonstrated previously^20,36,37^. Here we establish the precise molecular pathways by which the gut microbiota contributes to an antiviral response mediated by acetate production and induction of IFN-β in the lung with IFNAR engagement.

IFN-1 is normally secreted after virus recognition and binds to IFNAR 1 or 2, and initiates a signaling cascade that involves the JAK/STAT pathway and induces transcription of ISGs, which have direct antiviral activity^38^. IFNAR1 has a unique extracellular four-domain architecture comprised of subdomains SD1 to SD4, while IFNAR2 has only two^39^. The affinity of IFN-beta for the type I receptor is higher than other type 1 interferons being the most potent activator of the receptor. In agreement with a relevant role of IFNAR in the protective effect of acetate, we found that acetate treatment induced the transcription of well-characterized antiviral ISGs, *Oas1* (encodes oligoadenylate synthetase) and *Isg15* in the lungs of RSV-infected mice. The protein encoded by *Oas1* has been shown to decrease RSV replication^40^, a mechanism that may explain the acetate effects. The antiviral activity conferred by acetate treatment did not prevent RSV infection, as demonstrated by the presence of similar viral load in the lung of control and acetated-treated mice after 24 h of infection, but caused an impressive and consistent reduction in this parameter after longer periods (i.e., 72 and 120 h of infection).

The production of type I interferon during RSV infection must be carefully balanced to reduce viral load without allowing excessive cell infiltration in the lung. We found that acetate modulated the type 1 interferon response on lung epithelial cells, after oral and intranasal treatments. We hypothesize that this effect of acetate may be associated with a direct effect on epithelial cells that overcome the virus inhibitory mechanisms that act on the IFN-I pathway. RSV NS proteins have been shown to inhibit several pathways associated with IFN-β production and signaling including RIG-1^41^, IRF3^42^, STAT2^43^, while increasing miR-24, which inhibits the expression of IFNAR^44^. Alveolar macrophages are suggested to be the main source of type I interferon and are important to recruit inflammatory monocytes to the lung thus decreasing disease severity^34^. However, we did not find that macrophages produced type 1 interferon in response to acetate; the main source of IFN-b in our study was epithelial lung cells. Macrophages are described to be resistant to RSV replication, not resulting in the production of viral particles. This may lead to a reduced susceptibility in these cells to the effect of RSV NS proteins that are a natural inhibitor of type 1 response^45^. Goritzka, M. et al. 2015 suggested in their study that lung epithelial cells are not the major source of type 1 IFN even being the prevalent target of RSV infection due to the RSV NS protein blocking type 1 IFN production^34^. Acetate may have a direct effect on epithelial cells that overcome, at least in part, the virus inhibitory mechanisms of RSV NS proteins that act on the IFN-I pathway, and might be the reason why acetate presents its effects in epithelial cells and not in macrophages.

Consistent with our findings in *Ifnar−/−* mice, and confirming the importance of IFNAR on RSV infection, we found that the *IFNAR1* rs2257167 C allele (a valine to leucine substitution in the SD2 domain) was associated with protection against severe disease in RSV-positive infants. The IFNAR1 SD2 and SD3 interface contains a hinge center that mediates movement of the two halves upon ligand engagement^46,47^. Structural modeling revealed the larger leucine residue provides a stable conformation with better packing in the hydrophobic SD2 cavity^39^. While it does not reside on the SD2/3 interface, Leu168 may reposition critical amino acids in the ligand-binding hinge region that could alter IFNAR1 signaling. While we can only speculate as to the functional impact of this variant, infants with Leu168 may have altered IFNAR1 dynamics, either through improvement of ligand-binding or IFN-β recruitment, which confers protection from RSV disease. Importantly, the *IFNAR1* rs2257167 mutation is in strong linkage disequilibrium with proximal intronic and distal promoter SNPs associated with viral infection^39,48,49^. The Val168Leu substitution resides on the third beta strand of the IFNAR1 SD2 domain and has been associated with viral pathogens such as hepatitis B virus (HBV) and human immunodeficiency virus (HIV/AIDS)^39,49,50^. We also found that breastfeeding increased the genetic effect of the C allele with protection from severe disease. Although breastfeeding increased the levels of gut acetate in infants^51^, the involvement of short-chain fatty acids and diet on the genetic protection of *IFNAR1* polymorphism is the focus of future studies.

In addition to modulation of IFN-I production, acetate treatment reduced the recruitment of inflammatory cells including macrophages and lymphocytes to the lungs of mice, and reduced Th2 and dendritic cells activation in the lymph nodes after 5 days of RSV infection. This effect may be secondary to the reduction of viral replication and, in part, reflects the changes in inflammatory mediators, as observed for TNFα and IL-10 production in acetate-treated mice. These effects might be associated with the well-described pro-resolution effect of acetate^15^ and may partially explain the maintenance of the bronchoalveolar organization. ISG15 was associated with IL-10 production^52^, which can also contribute to the anti-inflammatory effect observed in our model. Furthermore, acetate and the other SCFAs, butyrate, and propionate have been described to have a role in activation and function of immune cells^8,13,53–55^.

Our data obtained with *Gpr43*^−/−^ mice characterized the participation of this receptor in the RSV response phenotypes. Acetate is known to activate GPR43, a receptor that is expressed in the membrane of different cells including epithelial cells^56^. This receptor was first characterized in the context of inflammatory conditions such as colitis^9^, in which its activation was shown to attenuate inflammation and to mediate the protective effects of SCFAs in the colon. Later, the role of this receptor in the context of metabolic and infectious diseases was also demonstrated^57–60^. Here we show the importance of GPR43 in the IFN-b production during RSV infection in mice treated with acetate. Trompette et al. 2018 demonstrated a role for the butyrate receptor, GPR41, during influenza infection^23^. Based on our findings, we hypothesize that acetate activates GPR43 in the lung, thus inducing IFN-β production and ISGs expression in the epithelial cells protection against RSV infection. We cannot exclude the possibility that oral administration of acetate also activates GPR43 in other tissues including the intestinal epithelial cells or immune cells, which may indirectly contribute to IFN-β production by pulmonary cells. A study demonstrated that NF-κB is one of the pathway mediated by GPR43^61^, and also NF-κB is an essential mediator of type 1 interferon response^28^. We identified that NF-κB is important to acetate to modulate IFN-β response in the lung epithelial cells through GPR43 engagement. In addition, we found greater susceptibility to RSV-induced disease phenotypes in strains of mice with the loss-of-function SNPs in *Ifnar1* and *Gpr43* compared to inbred strains of mice with the wild-type alleles. These results are consistent with a protective role for these two genes in the response to RSV infection in mice.

We found that HF-diet-fed mice had an increase of gut bacterial components of *Lachnospiraceae* spp, which are in a bacterial family usually isolated from human intestinal microbiota, including from infants in the first year of life^62^. A study showed a decrease in the *Lachnospiraceae* family following RSV infection in mice^63^. Increased abundance of this family has also been reported in other studies in which fermentable fiber was added, and was associated with increased serum levels of acetate^64^.

In conclusion, our data reveal a mechanism for protection against RSV infection in which acetate, a metabolite derived from the intestinal microbiota, induces IFN-β in the lung and the mechanism of protection is mediated by GPR43 and IFNAR. This mechanism might be also relevant for protection against other virus infection.

## Methods

### Virus

The RSV A2 strain was kindly provided by Dr. Fernando Polack, Fundación Infant, Argentina. Viral plaque-forming units (PFU) were identified using an anti-RSV antibody (Millipore, Billerica, MA, USA).

### Animals and RSV infection

Female BALB/c, male and female type 1 interferon receptor deficient (*Ifnar*−/−) 129/Sv, wild-type 129/Sv, female Rag-1 deficient (*Rag1−/−)*, female GPR43‐deficient (*Gpr43*^-/-^) and C57BL/6 mice, all at age 6–8 weeks, were used in the study. Mice were housed at the Animal Facility of the Institute of Biology, University of Campinas. All animal procedures were performed in accordance with protocols approved by Animals Ethics Committee of UNICAMP (protocols 4022–1 and 4599–1). For RSV infection, mice were anesthetized with 5% isoflurane and infected intranasally with 10^7^ PFU/mL of RSV A2 strain. All animals were weighed daily. Data analysis was performed 5 days post infection or as indicated. Bronchoalveolar lavage fluid (BALF) was collected and left lungs were removed after perfusion with formalin for Histopathological and Immunohistochemistry analysis.

### Diet and SCFA treatment

Mice were fed with high-fiber diet (HF) or control diet (CF) for 4 weeks before infection. Both diets were based on the AIN93M (American Society for Nutrition, USA) and detailed compositions are presented in Supplementary Table 5. In another experiment, high-fiber diet-fed mice received an antibiotic mix consisting of kanamycin (0.4 mg/mL), gentamicin (0.035 mg/mL), metronidazole (0.045 mg/mL), vancomycin (0.045 mg/mL), and colistin (0.035 mg/mL) diluted in the drinking water for 3 days before infection. All antibiotics were purchased from Sigma–Aldrich. Mice were given one of the SCFAs, sodium acetate, sodium propionate, or sodium butyrate (Sigma–Aldrich, St. Louis, MO, USA) in sterile drinking water at a final concentration of 200 mM for 3 weeks before the RSV infection and indicated in all experimental schematic representation. Another SCFA treatment was performed starting simultaneously with the infection at the same final concentration. Water volume consumption was measured daily and no difference in water intake was found between the groups.

### Bronchoalveolar lavage fluid (BALF)

Mice were anesthetized with intraperitoneal administration of ketamine (0.4 mg/g)/xylazine (0.2 mg/g) solution and the tracheas were cannulated. The lungs were washed twice with RPMI 1640 medium. BALF was centrifuged, the supernatant collected for cytokine analysis and pellets suspended for total cell and differential count and flow cytometry. For differential cell counting the cytospin slides was stained with May-Grunwald-Giemsa™ and the counting procedure was performed in a blinded manner by an experienced investigator.

### Histopathological and Immunohistochemistry analysis

The left lung was embedded in paraffin blocks, cut into 4-μm sections, stained with H&E or submitted for antigenic recovery and subsequent labeling with anti-RSV F protein antibody (Millipore, MA, USA). Slide analysis was performed in a blinded manner. The peribronchial and perivascular inflammation was scored according to Barends et al.^65^, as absent (0), minimal (1), slight (2), moderate (3), marked (4), or severe (5).

### Viral load quantification

Total lung RNA was extracted and complementary DNA (cDNA) was synthesized using GoScript reverse transcriptase (Promega). The amplification of the RSV F protein gene was performed using the indicated specific primers and probes: forward primer- 5’-AACAGATGTAAGCAGCTCCGTTATC-3’, reverse primer- 5’-GATTTTTATTGGATGCTGTACATTT-3’ and probe- 5’ FAM/TGCCATAGCATGACACAATGGCTCCT-TAMRA/-3’ by real-time PCR. Primer sequences were synthesized and cloned into pUC57 plasmids (GenScript, Piscataway, NJ, USA), to perform a 10-fold dilution and generate a standard curve for calculation of the viral load. The values obtained from viral copies (based on concentration of the plasmid control) were calculated relative to the weight of the pre-weighed lung portion (copies per gram of lung).

### Flow cytometry

Isolated cells from lung or axillary lymph nodes were incubated with Mouse Fc Block (#553141 BD Biosciences®) for 20 min and then stained with surface antibodies anti-CD11c (1:100, #561241, clone HL3), anti-I-Ad/I-Ed (1:200, #558593, clone 2G9), anti-CD86 (1:100, #553691, clone GL1), anti-CD8 (1:100, #561092, clone 53–6.7), anti-CD4 (1:200, #553046, clone RM4–5), anti-CD25 (1:100, #552880, PC61), and anti-CD62L (1:100, #553152, clone MEL-14) (BD Biosciences®). For intracellular staining, cells were fixed with cytofix/cytoperm (BD Biosciences®) and stained with anti-IL-4 (1:50, #562045, clone 11B11) (BD Biosciences®), anti-FoxP3 (1:50, #72–5775, clone FJK-16s), anti-IFNγ (1:50, #RM9001, clone XMG1.2) (eBioscience), and anti-IL-17a (1:50, #506939, clone TC11–18H10.1) (BioLegend) antibodies. Samples were analyzed on Gallios (Beckman Coulter®) and BD FACS Verse (BD Biosciences®) flow cytometers. Data were analyzed using the FlowJo software (version 10, Tree Star Inc., MA, USA).

### Microbiome analysis

Feces from mice fed with high-fiber or control diets and infected with RSV, were harvested under sterile conditions. Samples were stored at −80 °C. Bacterial DNA was isolated using the QiaAMP DNA Stool Mini Kit (Qiagen, Hilden, Germany) according to the manufacturer’s instructions. DNA was amplified using primers selected to cover the V4 region of bacterial 16 s rRNA (16 S Amplicon PCR Forward Primer = 5’ TCGTCGGCAGCGTCAGATGTGTATAAGAGACAGCCTACGGGNGGCWGCAG 16 S Amplicon PCR Reverse Primer = 5’ GTCTCGTGGGCTCGGAGATGTGTATAAGAGACAGGACTACHVGGGTATCTAATCC) with Phusion DNA Polymerase. Amplicons were purified with a magnetic bead capture kit (AMPure XP; Agencourt) using Nextera XT indices and normalized following the Illumina protocol. Sequencing of the 16 s rRNA gene marker was performed on a the Miseq platform with the v2 500 cycle kit (Ilumina, CA, USA), which generates paired-end reads (2 × 250 bp). The analytical pipeline incorporated phylogenetic and alignment based approaches to maximize data resolution. The read pairs were demultiplexed based on unique molecular barcodes added via PCR during library generation, then merged using USEARCH v7.0.1090^66^. The subsequent analysis steps leveraged custom analytic packages developed at the Alkek Center for Metagenomics and Microbiome Research (CMMR) at Baylor College of Medicine to produce summary statistics and quality control measurements. This enabled characterization of microbial communities across large numbers of samples and sample groups. 16 Sv4 rDNA sequences were clustered into Operational Taxonomic Units (OTUs) at a similarity cutoff value of 97% using the UPARSE algorithm. OTUs were subsequently mapped to an optimized version of the SILVA Database^67^ containing only sequences from the V4 region of the 16S rRNA gene to determine taxonomies. Abundances were recovered by mapping the demultiplexed reads to the UPARSE OTUs. A custom script was used to construct an OTU table from the output files generated in the previous two steps for downstream analyses using a visualization toolkit also developed at the CMMR (ATIMA*, Agile Toolkit for Incisive Microbial Analyses*). ATIMA is a stand-alone tool for analyzing and visualizing microbiome data sets. This software provides an integrated solution for exploring relationships between microbial communities and emergent properties of their hosts or environments^68,69^.

### SCFAs quantification

Colonic luminal content samples were harvested from mice and used for measurement of SCFAs, following a protocol similar to that used by Fellows et al.^70^ Samples were weighted, crushed, and homogenized in water. Sodium chloride, citric acid, hydrochloric acid, and butanol were added to the samples, which were vortexed and centrifuged and the supernatants collected. A calibration curve with 0.015–0.1 mg/mL SCFAs was used in the quantification. Chromatographic analyses were performed using a gas chromatograph-mass spectrometer (model GCMS-QP2010 Ultra; Shimadzu) and a fused-silica capillary Stabilwax column (Restec Corporation, USA) with dimensions of 30 m × 0.25 mm internal diameter (i.d.) and coated with a 0.25-µm thick layer of polyethylene glycol. Samples (1 µL) were injected at 250 °C using a split ratio of approximately 25:1. High-grade pure helium (He) was used as the carrier gas with a constant flow rate of 1.0 mL/min. Mass conditions were as follows: ionization voltage, 70 eV; ion source temperature, 200 °C; full scan mode in the 35–500 mass range with 0.2 s/scan velocity. The runtime for each analysis was 11.95 min.

### Pulmonary cell sorting

Left lung from mice were harvested 72 h after RSV infection and acetate treatment. The lung was immediately submitted to collagenase IV (1 mg/ml) digestion protocol and total cells were stained with anti-mouse CD45 FITC (1:200, #553080, clone 30-F11) (BD Biosciences®) and anti-mouse CD326 (EpCAM) PE-Cy7 (1:100, #25–5791–80, clone G8.8) (eBioscience) antibodies for 30 min. Cells were acquired on BD FACSAria flow cytometer (BD Biosciences®) and sorted in two different populations: CD45^+^CD326^−^ (leukocytes/lymphoid cells) and CD45^−^CD326^+^ (epithelial cells). Right after the separation of the populations, cells were centrifuged, suspended in TRIzol (Thermo Fisher Scientific) and processed for RNA extraction and cDNA synthesis. The *Ifnb1* expression was made as described below.

### Type 1 interferon analysis

Lung cDNA (synthetized as described above) was used to determine IFN-α and IFN-β relative expression levels using primers and probes for TaqMan Assay (Mm03030145_gH *Ifna1*, Mm00439552_s1 *Ifnb1* Thermo Fisher Scientific) and mouse β-actin (Mm02619580_g1 Actb) as an endogenous control gene. ISG expression was accessed using the following primer sets: *Oas1* (forward primer: 5’-AAAAGGAGGAGCCATGGCAGT-3’ reverse primer: 5’-CTGAGCCCAAGGTCCATCAG-3’); *Isg15* (forward primer: 5’-GAGCTAGAGCCTGCAGCAAT-3’ reverse primer: 5’-TCACGGACACCAGGAAATCG-3’); and *B2M* (forward primer: 5’-CCCCAGTGAGACTGATACATACG’ reverse primer: 5’-CGATCCCAGTAGACGGTCTTG’). PCR conditions followed the GoTaq™ Probe qPCR Master Mix (Promega™, Madison, WI, USA) or SYBR Green/ROX qPCR Master Mix protocols (Thermo Fisher Scientific, Waltham, MA, USA). Quantification of gene expression was conducted using StepOne™ (Applied Biosystems). The ΔΔCt or 2^-ΔCt^ analysis was used to calculate de gene expression. IFN-α and IFN-β protein levels were quantified in BALF supernatants using an IFN alpha/IFN beta 2-Plex Mouse ProcartaPlex™ Panel assay kit (Invitrogen, Thermo Fisher Scientific, Waltham, MA, USA). Assays were performed according to manufacturer’s instructions and read on the MAGPIX b350 Multiplexing Instrument (Merck Millipore, Billerica, MA, USA).

### Cell lines

Human adenocarcinoma alveolar epithelial cells (A549- ATCC CCL-185), human embryonic lung fibroblasts (MRC-5 ATCC CCL-171), epithelial cells extracted from kidney of African green monkey (Vero ATCC CCL-81) and A549 *Ifnar1*^−/−^ cells kindly provided by Dr. Daniel Mansur were cultured in DMEM low glucose (Gibco™, Thermo Fisher Scientific, Waltham, MA, USA) both supplemented with 10% of heat inactivated fetal bovine serum (FBS) (Cultilab, Campinas, SP, Brazil). All the cells were tested for mycoplasma contamination.

### In vitro SCFA treatment

Vero, MRC-5, and A549, and A549 *Ifnar1*^−/−^ cells were pre-treated with sodium acetate (Sigma–Aldrich, St. Louis, MO, USA) at different concentrations (60, 120, 200, and 260 μM) for 6, 12, and 24 h in DMEM containing 10% FBS and the cells were infected with 10^4^ PFU/mL of RSV in fresh medium. Alternatively, the cells were treated with SCFA 2 h after infection with RSV. After 96 h cell viability was determined using the MTT assay or propidium iodide (PI) staining using flow cytometry. Viral loads were measured by real-time PCR (as described above) and viral plaque assay. Additionally, MRC-5 and A549 were pre-treated with sodium propionate and butyrate similarly as described above.

### Western blotting analysis

A549 cells, cultured in six-well plates, were pre-treated with acetate at 260 μM for 24 h and then infected with 10^4^ PFU/mL of RSV for 2 h. Cells were harvested and cytoplasmic protein was extracted using an specific buffer (10 mM HEPES; pH 7.5, 10 mM KCl, 0.1 mM EDTA, 1 mM dithiothreitol (DTT), 0.5% Nonidet‐40, and 0.5 mM PMSF along with the protease inhibitor cocktail) followed by nuclear protein extraction (20 mM HEPES (pH 7.5), 400 mM NaCl, 1 mM EDTA, 1 mM DTT, 1 mM PMSF with protease inhibitor cocktail). Protein samples were separated on 10% SDS-page polyacrylamide gels and transferred to nitrocellulose membrane (Bio-rad). Later the membrane was stained with NFκB p65 (F-6) monoclonal antibody (1:300, #sc-8008, Santa Cruz Biotechnology), mouse β-actin monoclonal antibody (1:2000, #A2228, Sigma–Aldrich) or mouse PCNA monoclonal antibody (1:250, #NCL-L-PCNA, Novocastra) followed by secondary staining with 1:1000 of rabbit anti-Mouse IgG (H + L)-HRP antibody (#61–6520, ThermoFisher Scientific). Membrane blocking steps and antibody dilutions were performed using 5% (v/v) skim milk in 1X PBS, and washing steps performed with Tween-20. Western blots were visualized by enhanced chemiluminescence (GE Lifescience). For the quantification of the bands, ImageJ software was used. β-actin (cytoplasmic protein) and PCNA (nuclear protein) were used to normalize protein quantification. The blot uncropped version is available in Source Data file.

### Viral plaque assay

A549 and MRC-5 cells, cultured in six-well plates, were pre-treated with acetate at 260 μM for 24 h and then infected with 10^4^ PFU/mL of RSV for 96 h. Cells were harvested, submitted to freeze-thaw and then inoculated onto Vero cell monolayers. Viral PFUs were determined using an anti-RSV (1:1000, #AB1128, Millipore, Billerica, MA, USA) antibody after 4 days.

### Mouse primary cell culture

To obtain pulmonary cells, left and right lung from naïve mice were harvested, The pieces were cut into cubic pieces and subjected to enzymatic digestion with 1X trypsin for 1 hour at 37 °C. After complete digestion, cells were cultured with 10% FBS DMEM medium for 24 h, and then the culture medium was exchanged for a fresh one for another 48 h. All experiments were performed between the first and second passages of the cells. To obtain mouse alveolar macrophages cells from BAL of acetate-treated mice were collected and incubated for 2 h for macrophage adhesion then macrophages were infected with RSV or infected and further treated in vitro with acetate (260 µM). After 24 h, the culture supernatants were analyzed for IFN-β production.

### Statistical analysis

All in vitro experiments were performed in triplicates. In vivo experiments were performed at least two times (*n* = 3–9 mice per group in each experiment). Kruskal–Wallis test was applied to determine statistical significance between the various experimental groups and Mann–Whitney was applied to compare two experimental groups using GraphPad Prism software (San Diego, CA, USA). Values presented in graphs are the mean ± standard error of mean (SEM) and a *p* < 0.05 was considered significant. Associations between *Ifnar1* and *Gpr43* (*Ffar2*) genotypes and phenotypes (i.e., allelic group means) within mouse strains using two-tailed Student’s *t*-test. We tested only non-synonymous coding SNPs.

### Human population

A case-control study was conducted in Buenos Aires, Argentina (2003 to 2006) with healthy full-term infants (<1 year of age) presenting with bronchiolitis. Diagnosis of RSV infection was performed by trained pediatricians based on clinical outcomes (oxygen saturation, Th2 polarization, RSV titer and pCO_2_), and only RSV-positive subjects (246 severe and 172 mild) were included for the study. The selection criteria for RSV disease severity was based on a clinically relevant endpoint: healthy full-term infants with bronchiolitis were recruited to the study if their oxygen saturation upon enrollment was lower than 93% when breathing room air. Exclusion criteria included known or suspected impairment of immunological function, major congenital oral malformations, chronic lung disease, cardiac disease, prematurity (gestational age of less than 37 weeks), neuromuscular disorders affecting swallowing, and known or suspected coagulation disorders or bleeding tendency^29^. A total of 17 samples were excluded from genetic analyses due to no signal or other problems with the DNA. The Institutional Review Boards of the French Hospital (Buenos Aires) and the network of Hospital Materno Infantil de San Isidro (which encompasses the three hospitals in the northeastern region of Buenos Aires), as well as the Institutional Review Board of Johns Hopkins University (Baltimore, MD, USA), approved the protocol, and the study conformed to standards indicated by the Declaration of Helsinki. Informed consent was obtained from the parents of each enrolled infant.

### DNA extraction and genotyping for *IFNAR1* rs2257167

All the data were collected during the study performed in 2003–2006. We queried the NIEHS TagSNP database to identify a *IFNAR1* tagSNP that provided adequate gene coverage (Supplementary Fig. 7). DNA was extracted from whole blood using the Gentra PureGene kit (Gentra Systems, Minneapolis, MN, USA), characterized for purity and concentration, and a 250 ng aliquot submitted for genotyping using the Illumina BeadXpress assay. Briefly DNA was activated and labeled with a Cy3 or Cy5 tag, hybridized to validated *IFNAR1* sequence-specific paramagnetic VeraCode beads (reverse strand: 3’-cttctacacctgaagagtttttccagataa [C/G] taagctatatgtaaagcttaaaccatccaa-5’), and the fluorescent signal scanned using the BeadXpress system. Clusters that passed separation and SNP call frequency were refined by additional quality control measures and analyzed using Genome Studio. Genotype calls were exported to Excel and ASCII coded for association analysis. Genotyping for *IFNAR1* rs2257167 was confirmed by allelic discrimination using a validated TaqMan SNP genotyping assay (Fisher Scientific, MA, USA). Five percent of samples were submitted for sequencing and calls were 100% concordant. The phenotype of interest was clinically defined as “severe” or “mild” RSV disease and served as a dichotomous dependent variable in our statistical model. To determine whether or not the *IFNAR1* tagSNP associated with RSV disease severity, we formulated three hypotheses: (1) the additive effect model, which contained all three genotypes: GG, GC, and CC as levels of the predictor variable; (2) a dominant effect model of variant allele, in which the predictor variable had two levels, corresponding to the GG vs. GC/CC genotypes combined; (3) a recessive effect model of variant allele, in which the predictor variable had two levels, which corresponded to GG/GC combined vs. CC genotypes. For each of these three hypotheses, we performed logistic regression and for the dominant/recessive effect models, we derived the odds ratio of the genotype effect and its corresponding 95% confident interval for each estimate. In addition to the base logistic model, we also tested other factors including gender, breastfeeding, region/hospital location, and socioeconomic status as covariates in the logistic model. All tests and estimates were made and adjusted for the covariate accordingly.

### Reporting summary

Further information on research design is available in the Nature Research Reporting Summary linked to this article.

## Supplementary information

Supplementary Information

Reporting Summary

Source Data

## Data Availability

The source data underlying Fig. 1b, c, d, g, h, j; 2b, c, d, f; 3b-d; 4b, c, d, g, i, j; 5a-d; 6a, b, c, e, f, g, h, I, k, m; 7a, b, c, d, f, h, i, j; 8b-d; 9a, b, c, f, g, i; and Supplementary Fig. 5a, d, e, f, 6 a, b and d, 7, 8 a and c are provided in the Source Data file. All other relevant data are available from corresponding authors upon reasonable request. The script used to run 16 S analysis at the CMMR is available on GitHub: CMMR-16S rbiom.
