# Supplementary material for: Microbiota-derived acetate protects against respiratory syncytial virus infection through a GPR43-type 1 interferon response
Source: Nat Commun. 2019 Jul 22;10:3273. doi: 10.1038/s41467-019-11152-6 (PMC6646332; doi:10.1038/s41467-019-11152-6)
Supplement: Supplementary file 1 — Supplementary Information [file 41467_2019_11152_MOESM1_ESM.pdf]

Supplementary information

Microbiota-derived acetate protects against respiratory syncytial virus infection through a GPR43-type 1 interferon response

Antunes et al.

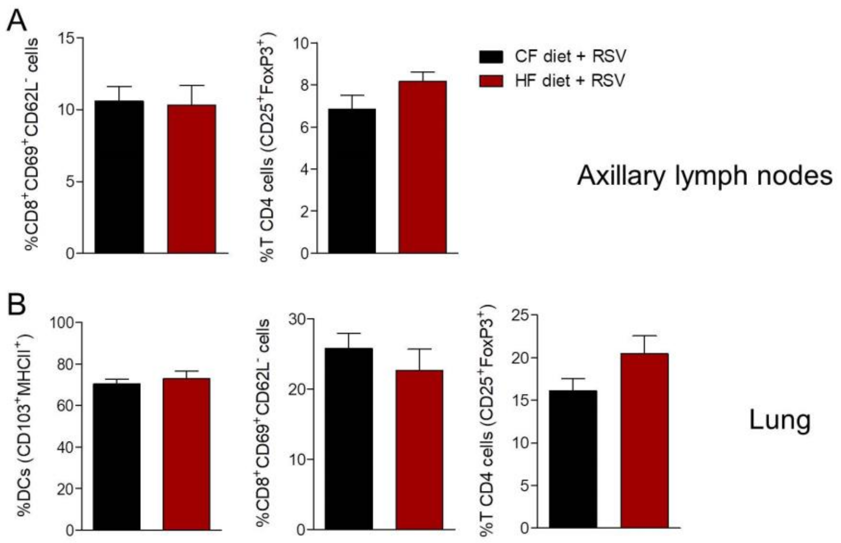

Supplementary Figure 1. Analysis of different immune cells in animals treated with control and high-fiber diet and infected with RSV. A, Percentage of activated T CD8 cells (CD8+CD69+CD62L-), and T regulatory cells (CD3e+CD4+CD25+FoxP3+) in the axillary lymph nodes. B, Percentage of tissue resident dendritic cells (CD11c+CD11b-MHCII+CD103+), activated T CD8 cells and T reg cells in the lung. All data are expressed as mean ± SEM (n= 4-6 mice per group). The statistical significance was determined by Mann-Whitney test. *p < 0.05, **p < 0.01, ***p < 0.001.

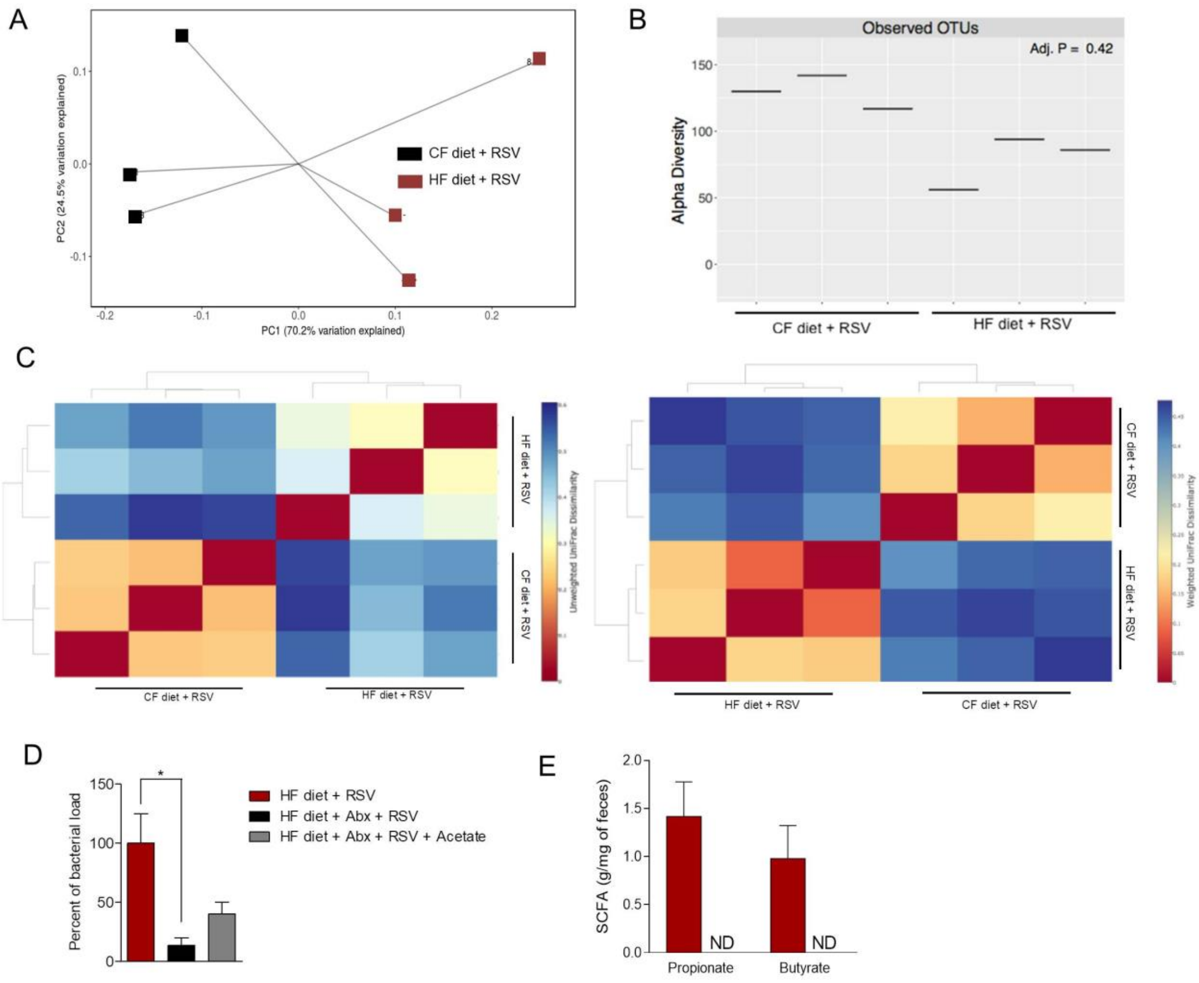

Supplementary Figure 2. HF diet alters intestinal microbiota. A, Analysis of the variance between microbial communities from fecal samples of mice fed with both diets (control and high fiber) assessed by the average relative abundance using principal component (PC) analysis. B, Alpha diversity (in number of operational taxonomic units (OTUs)) in feces of animals from CF and HF groups. C, Weighted and unweighted UniFrac dissimilarity analysis of bacterial communities in feces. D, Percent of bacterial load in fecal samples of animals that received high-fiber diet and were treated or not with antibiotic mix (Abx). Analysis was performed by real time PCR. E, Propionate and butyrate quantification in fecal samples of animals that received high-fiber diet and were treated or not with Abx. Data in D are expressed as mean ± SEM (n= 4-6 mice per group). Data in A-C are representative of 3 animals. The statistical significance was determined by using two-way ANOVA followed by Bonferroni post hoc test. *$p < 0.05$.

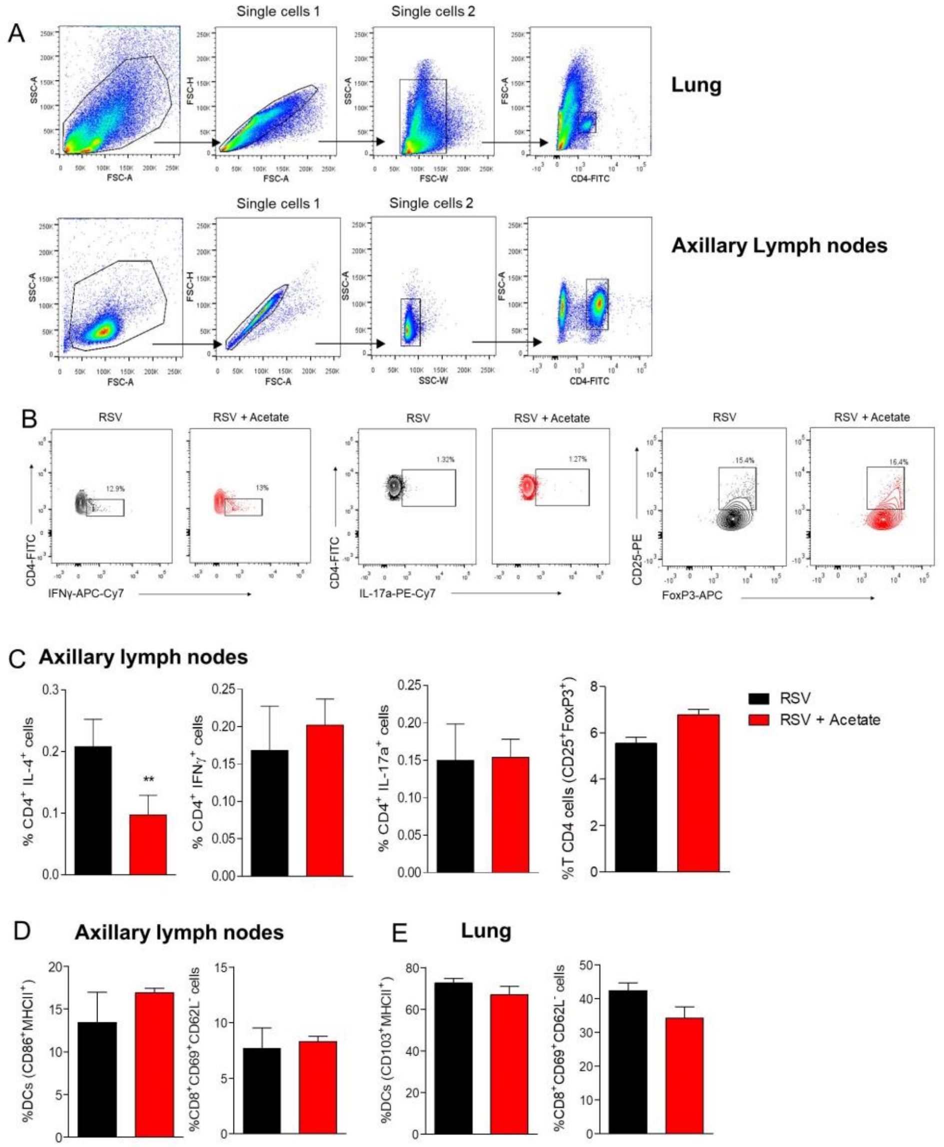

Supplementary Figure 3. Analysis of different immune cells in animals treated with acetate and infected with RSV. A, Gate strategy of CD4 T cell population in flow cytometry analysis. B, Representative FACS profile of flow cytometry data showed in Figure 4H. C, Percentage of different T cell-producing cytokines and T regulatory cells (CD3e+CD4+CD25+FoxP3+) in axillary lymph nodes. D, Percentage of activated dendritic cells (CD11c+CD11b+MHCII+CD86+) and activated T CD8 cells (CD8+CD69+CD62L-) in the axillary lymph nodes. D, Percentage of tissue resident dendritic cells (CD11c+CD11b-MHCII+CD103+) and activated T CD8 cells and in the lung. All data are expressed as mean ± SEM (n= 5 mice per group). The statistical significance was determined by Mann-Whitney. *p < 0.05, **p < 0.01, ***p < 0.001.

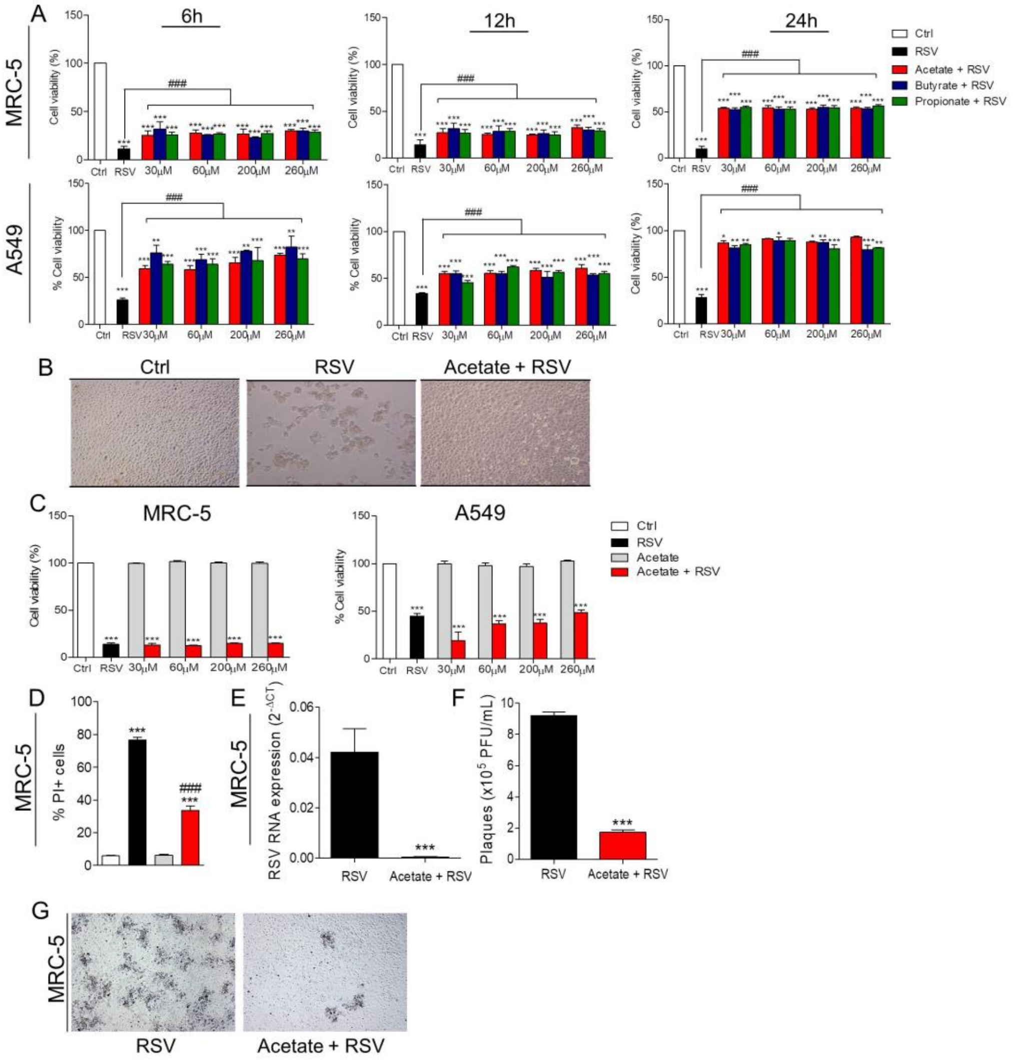

Supplementary Figure 4. **Acetate pretreatment protects against cell death caused by RSV.** A, MRC-5 and A549 cells were pretreated with acetate, propionate and butyrate at different concentrations (30, 60, 200 and 260uM) during 6h, 12h, or 24h and then infected with RSV. Cell viability was assessed by MTT assay after 96h. B, Representative microscopic images of A549 cells pretreated with acetate and infected with RSV. C, MRC-5 and A549 cells were infected with RSV and after 2h treated with acetate at different concentrations. Cell viability was assessed by MTT assay after 96h. D, Percentage of PI (propidium iodide) positive cells detected by flow cytometry. E, RSV RNA levels detected using real-time PCR (2-ΔCt analysis). F, Quantification of RSV plaque-forming units (PFU) detected by viral titration protocol. G, Representative images of viral title assay indicating the viral plaques. Lysis plate titration was performed using an anti-RSV antibody. Data are shown in triplicates of 2 independent experiments. * represents significant difference relative to ctrl; # represents significant difference relative to RSV. All results are expressed as mean ± SEM. Statistical significance was determined with Kruskal-Wallis, except in E and F which Mann-Whitney was used.*p < 0.05, **p < 0.01, ***p < 0.001.

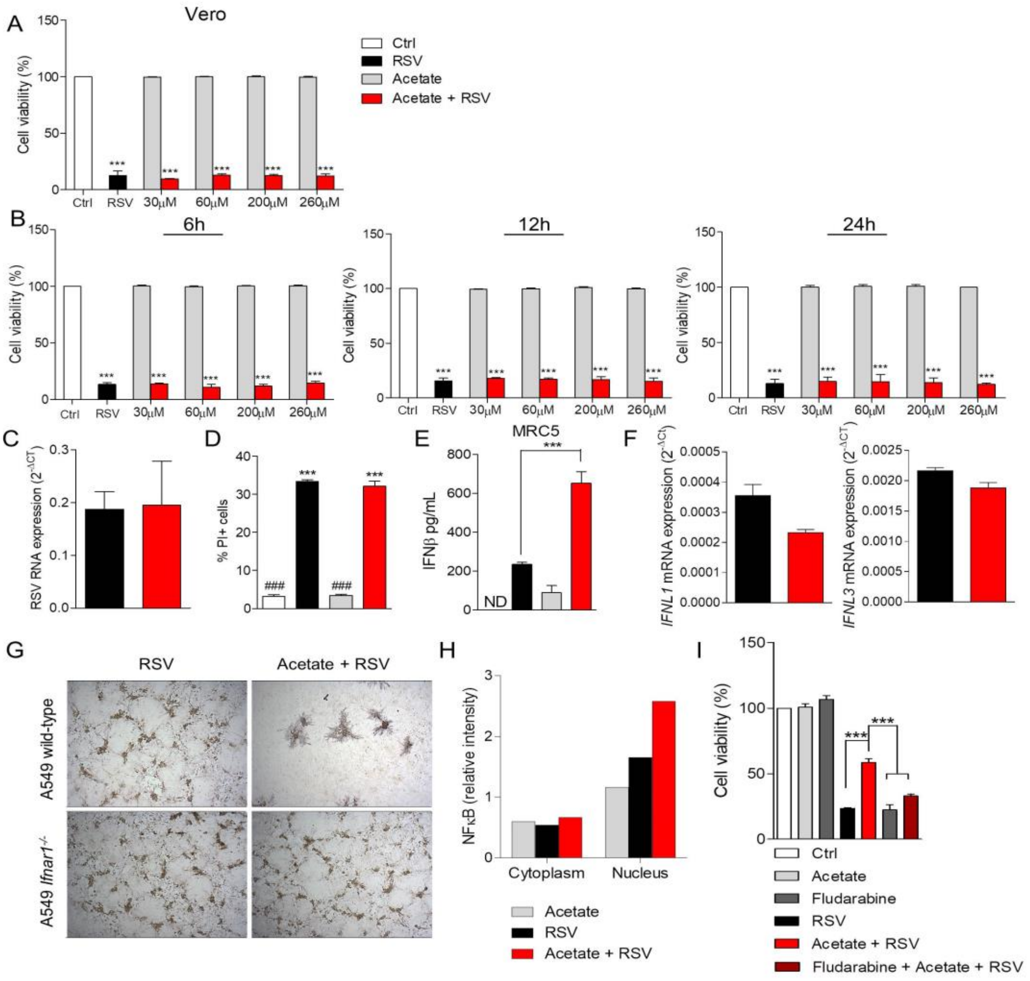

Supplementary Figure 5. Acetate protection against RSV *in vitro* depends on IFNAR presence. A, Vero cells were infected with RSV and after 2h treated with acetate at different concentrations. Cell viability was assessed by MTT assay after 96h. B, Vero cells were pretreated with acetate at different concentrations and times and infected with RSV. Cell viability was assessed by MTT assay after 96h. C, RSV RNA levels detected in Vero cells pretreated during 24h with acetate (260 µM) before RSV infection. Real time PCR analysis performed after 96h. D, Percentage of cell death in Vero cells pretreated 24h with acetate and infected with RSV for 96h. E, IFN-β protein levels on supernatant of MRC-5 cells pretreated with 260 µM of acetate for 24h and infected with RSV for additional 24h. F, A459 cells were treated with acetate (260 µM) for 24h and infected with RSV (10^4 PFU/ml) for another 24h and analyzed the expression of *IFNL1* and *IFNL3* (interferon lambda genes) by real-time PCR. G, Representative images of RSV plaque-forming assay from A549 WT and A549 *Ifnar1-/-* pretreated with acetate for 24h and infected with RSV for 96h. Lysis plate titration was performed using an anti-RSV antibody. H, Quantification of NF-κB p65 by western blot analysis. Band intensity was normalized by its control protein (β-actin or PCNA). I, A549 cells were pre-treated for 1h with 15 µM of Fludarabine phosphate (an inhibitor which causes a specific depletion of STAT1 protein). Afterwards, cells were then treated with acetate (260 µM) for 24h and then infected with RSV. Cell viability was evaluated by MTT assay. * represents significant difference relative to ctrl; # represents significant difference relative to RSV. All results are expressed as mean ± SEM. Statistical significance was determined with Kruskal-Wallis, except on C and F which Mann-Whitney was used. .*$p < 0.05$, **$p < 0.01$, ***$p < 0.001$.

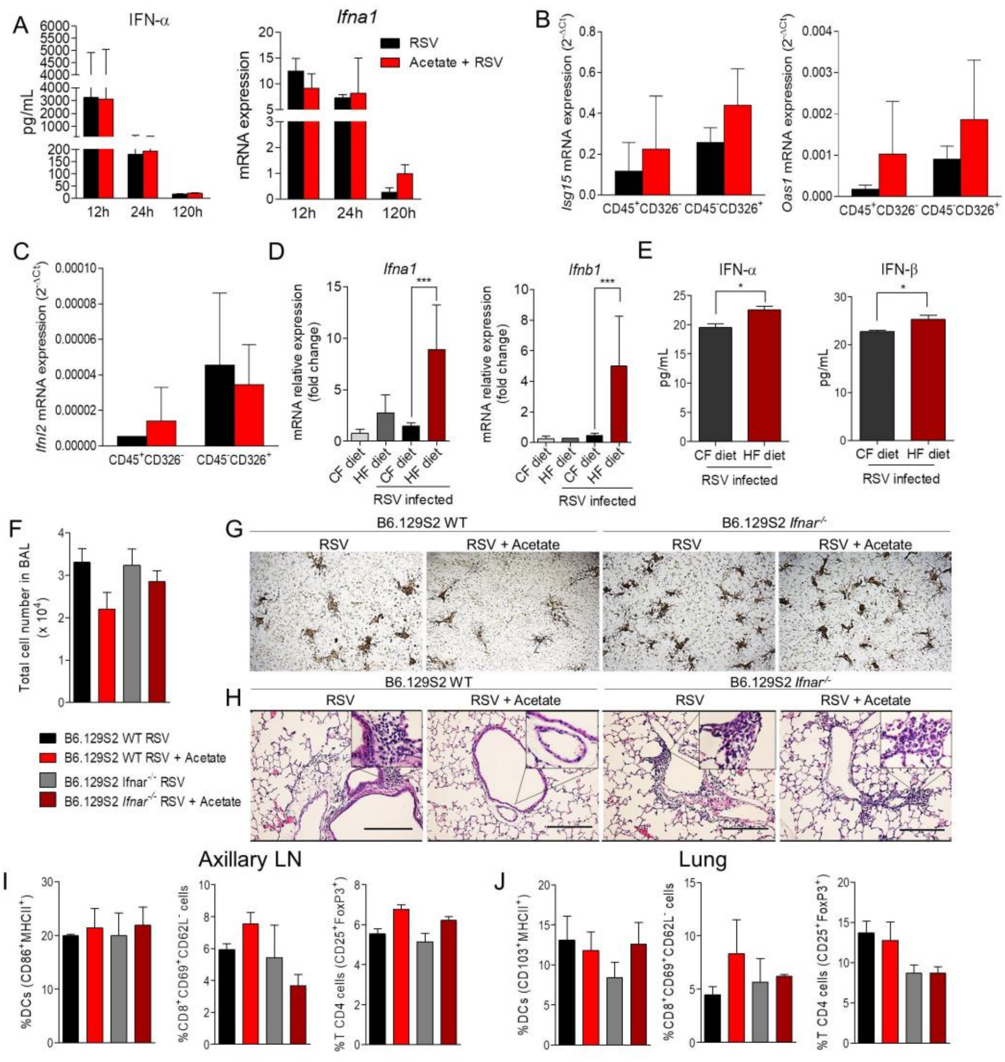

Supplementary Figure 6. Acetate protection against RSV infection *in vivo* depends on IFNAR presence. A, Female Balb/c mice were treated with acetate (200 mM) in drinking water and infected with RSV (10^7 PFU/ml) for 12h, 24h and 120h. A, IFN-α protein level in BAL and *Ifna1* gene expression in the lung. B and C, *Isg15*, *Oas1* and *Ifnl2* expression of CD45+CD326- and CD45-CD326+ sorted cells from lung of mice acetate treated and RSV infected for 72h (n = 5). D, mRNA expression of *Ifna1* and *Ifnb1* genes in the lung of animals fed with control (CF) or HF fiber diet (during 3 weeks) and infected with RSV. Data were accessed by real-time PCR on day 5 post infection. E, IFN-α and IFN-β protein detected in the BAL supernatant on day 5 post infection. F-J, Wild-type and *Ifnar-/-* mice were simultaneously infected with RSV and treated with acetate (200 mM) in drinking water. Analyses were performed at day 5 post infection. F, Total cell number in BAL. G, Representative images of the viral titration assay performed in PFU. H, Representative images of lung tissue sections stained with H&E. Scale bar=100 µm. I, Percentage of activated dendritic cells (CD11c+CD11b+MHCII+CD86+), activated T CD8 cells (CD8+CD69+CD62L-), and T regulatory cells

(CD3e+CD4+CD25+FoxP3+) in the axillary lymph nodes. J, Percentage of tissue resident dendritic cells (CD11c+CD11b-MHCII+CD103+), activated T CD8 cells, and T reg cells in the lung. All results are expressed as mean ± SEM (n= 4-6 mice per group, except in F, which 3 animals were used). Statistical significance was determined with Kruskal-Wallis, except in E, in which was used Mann-Whitney test. *$p < 0.05$, **$p < 0.01$, ***$p < 0.001$.

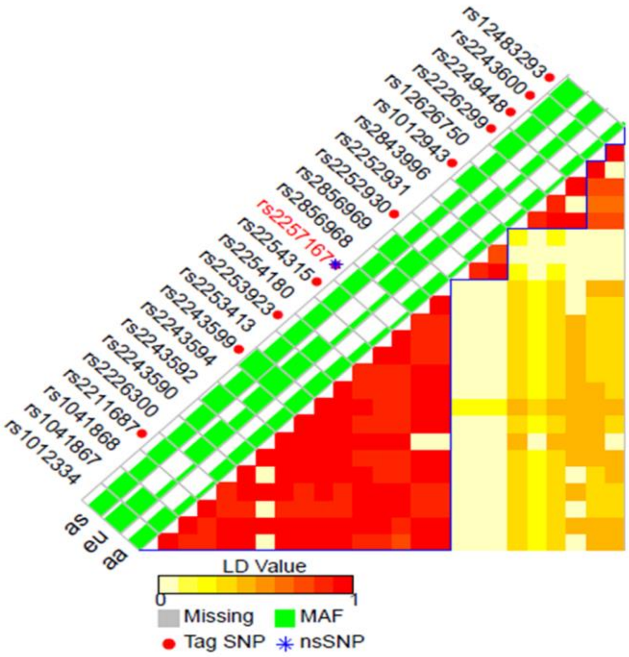

Supplementary Figure 7. Linkage disequilibrium for the IFNAR1 rs2257167 SNP was calculated using NIEHS TagSNP for Asian, European and African American HapMap populations. (https://snpinfo.niehs.nih.gov/snpinfo/snptag.html).

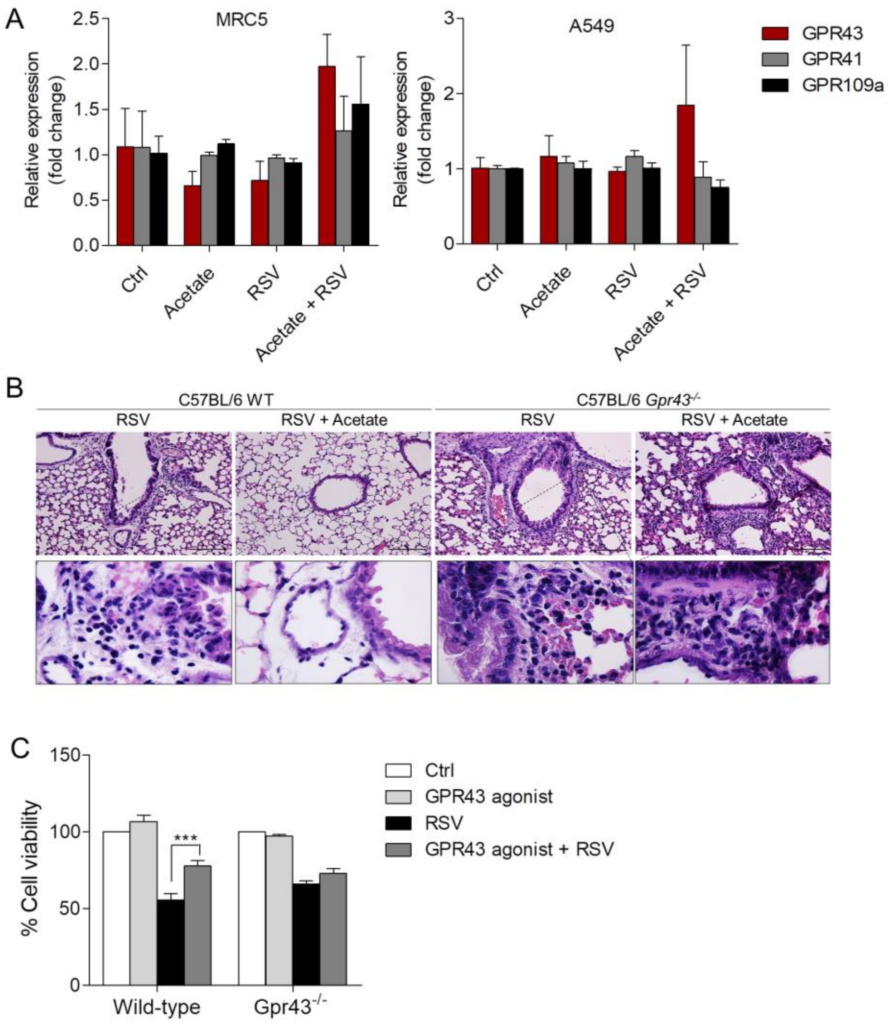

Supplementary Figure 8. Acetate effect relies on GPR43 activation. A, mRNA expression of *Gpr43, Gpr41* and *Gpr109a* in MRC-5 and A549 cells pretreated with 260 µM of acetate for 24h and infected with RSV for 24h. B, Representative images of lung tissue sections stained with H&E from *Gpr43* knockout mice and their controls (WT) simultaneously infected with RSV and treated with acetate in drinking water (200 mM). Analyses were performed at day 5 post infection. Scale bar=100 µm. C, Pulmonary epithelial cells from female C57BL/6 wild-type and Gpr43-/- mice were treated with acetate (260 µM) for 24h and then infected with RSV (10^4 PFU/ml) for 4 days. Cell viability evaluated by MTT assay. Data are expressed as mean ± SEM and shown in triplicates or 3 animals (B). Statistical significance was determined with Kruskal-Wallis. *$p < 0.05$, ** $p < 0.01$.

A

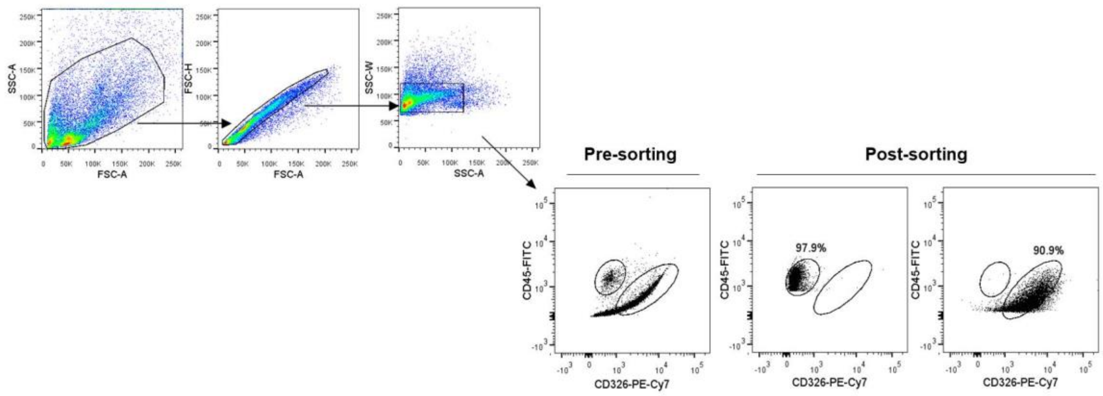

Supplementary Figure 9. A, Gate strategy of sorted CD45+CD326- and CD45-CD326+ population in lung tissue. Representative FACS profile of flow cytometry data showed in Figure 7 F.

Supplementary Table 1 - Relative abundance of the fecal microbiota composition at the family level

Family	CF diet + RSV	HF diet + RSV	95% CI	*p* Value
Lachnospiraceae	26,54	51,07	17.39 to 31.67	<0.001
Bacteroidaceae	32,93	30,34	-9.728 to 4.554	> 0.05
Ruminococcaceae	9,757	5,210	-11.69 to 2.594	> 0.05
Helicobacteraceae	8,563	1,580	-14.12 to 0.1576	> 0.05
Rikenellaceae	4,557	1,167	-10.53 to 3.751	> 0.05
Enterobacteriaceae	0,9367	2,460	-5.618 to 8.664	> 0.05
Erysipelotrichaceae	2,403	0,9833	-8.561 to 5.721	> 0.05
Verrucomicrobiaceae	2,507	0,0000	-9.648 to 4.634	> 0.05

CF, control fiber; HF, high fiber; RSV, respiratory syncytial virus; CI, confidence interval.

Supplementary Table 2. Allele frequency assessment in our population*

	Reference allele (G) frequency	Variant allele (C) frequency
**ALL individual (737)**	0,7313	0,2687
**Individual with RSV positive (401)**	0,7319	0,2681

*** In our population, 737 individuals were genotyped. Among these individuals, 401 were tested positive for RSV infection. The allele frequencies were calculated both groups.**

Supplementary Table 3 - Logistic regression tests for association between SNP genotypes and disease outcome.*

Gene	dbSNP ID	Reference	Variant	Genotype	Num_of_individual
IFNAR1	rs2257167	G	C	GG	219
				GC	149
				CC	33

Gene	dbSNP ID	Reference	Variant	Test Case	Genotype	Hypothesis	p value	Odds ratio	95% C.I. of odds ratio
IFNAR1	rs2257167	G	C	Case I	GG, GC, CC	Additive effect	0,0016		
				Case II	GG vs. GC/CC	Variant dominant effect	0,0110	0,5932	0.3966, 0.8874
				Case III	GG/GC vs/ CC	Variant recessive effect	0,0061	0,3549	0.1694 , 0.7437

Gene	dbSNP ID	Reference	Variant	Test Case	Genotype	Hypothesis	Covariate	p value	Odds ratio	95% C.I. of odds ratio
IFNAR1	rs2257167	G	C	Case I	GG, GC, CC	Additive effect	Gender	0,0022		
				Case II	GG vs. GC/CC	Variant dominant effect		0,0138	0,6019	0.4019, 0.9015
				Case III	GG/GC vs/ CC	Variant recessive effect		0,0082	0,367	0.1745, 0.7720

Gene	dbSNP ID	Reference	Variant	Test Case	Genotype	Hypothesis	Covariate	p value	Odds ratio	95% C.I. of odds ratio
IFNAR1	rs2257167	G	C	Case I	GG, GC, CC	Additive effect	Region	0,0017		
				Case II	GG vs. GC/CC	Variant dominant effect		0,0114	0,5938	0.3966, 0.8891
				Case III	GG/GC vs/ CC	Variant recessive effect		0,0068	0,3593	0.1711, 0.7542

Gene	dbSNP ID	Reference	Variant	Test Case	Genotype	Hypothesis	Covariate	pvalue	Odds ratio	95% C.I. of odds ratio
IFNAR1	rs2257167	G	C	Case I	GG, GC, CC	Additive effect	Breastfeeding	0,0014		
				Case II	GG vs. GC/CC	Variant dominant effect		0,0098	0,5867	0.3916, 0.8791
				Case III	GG/GC vs/ CC	Variant recessive effect		0,0054	0,349	0.1662, 0.7328

Gene	dbSNP ID	Reference	Variant	Test Case	Genotype	Hypothesis	Covariate	pvalue	Odds ratio	95% C.I. of odds ratio
IFNAR1	rs2257167	G	C	Case I	GG, GC, CC	Additive effect	SocEcoStat	0,0016		
				Case II	GG vs. GC/CC	Variant dominant effect		0,0106	0,5911	0.3950, 0.8846
				Case III	GG/GC vs/ CC	Variant recessive effect		0,0066	0,3583	0.1709, 0.7515

* S2. A. The number of individuals with each genotypes (GG, GC or CC). (B-F). Association test results. For each setting, we tested three hypotheses to explain the (1) additive effect (2) dominate effect and (3) recessive effect from the SNP rs2257167. Table S2 C-F were results from the similar logistic model with gender, region/hospital location, breastfeeding, and social economic status as a covariate.

Supplementary Table 4 - Functional single nucleotide polymorphisms (SNPs) in Ifnar1 and Gpr43 (Ffar2) that associate with RSV-induced lung inflammation phenotypes in inbred strains of mice. Phenotypes represent means (± SEM) for inbred strains that are homozygous for either the major or minor allele for each of the SNPs.

Gene/SNP rs ID	BP Position	SNP Function	Phenotype	Major allele, mean ± SEM	Minor allele, mean ± SEM	P-value
***Ifnar1* rs31418313**	91499433	ARG274His	PMNs	A, 155.3 ± 19.8	G, 280.6 ± 96.7	0.0314
			BW change (%)	A, 1.24 ± 0.88	G, -2.1 ± 0.95	0.0223
***Gpr43* rs47500117**	30819243	Ile291Leu	Protein	T, 297.8 ± 25.9	G, 456.6 ± 88.0	0.0159
***Gpr43* rs236386097**	3081922	Met298Val	Total cells	T, 355.2 ± 23.4	C, 478.8 ± 82.9	0.0416
			Monocytes	T, 16.5 ± 3.9	C, 44.9 ± 7.7	0.0009
			Protein	T, 290.5 ± 29.3	C, 475.1 ± 70.1	0.0051
			Mucus	T, 0.69 ± 0.08	C, 1.47 ± 0.54	0.0181

PMNs, polymorphonuclear leukocytes; BW, body weight; BP, base pair position for SNPs in *Infar1* (chromosome 16) and Gpr43 (chromosome 7)

Supplementary Table 5 – Diet componentes

	Control diet (g)	High-fiber diet (g)	Zero-fiber diet (g)
**Cornstarch, Rhoster®**	465.7	465.7	515.7
**Protein (casein), Rhoster®**	140	140	140
**Dextrinized starch, Rhoster®**	155	55	55
**Sucrose, Rhoster®**	100	100	100
**Soybean Oil, Liza®**	40	40	40
**Pectin, Vetec®**	0	100	0
**Celulose, Rhoster®**	50	50	0
**Mineral Mix (AIN 93M), Rhoster®**	35	35	35
**Vitamin Mix (AIN 93M), Rhoster®**	10	10	10
**L-Cystine, Rhoster®**	1.8	1.8	1.8
**Coline Bitartrate (41% coline), Rhoster®**	2.5	2.5	2.5
