# Supplementary material for: Microbiota-derived acetate protects against respiratory syncytial virus infection through a GPR43-type 1 interferon response
Source: Nat Commun. 2019 Jul 22;10:3273. doi: 10.1038/s41467-019-11152-6 (PMC6646332; doi:10.1038/s41467-019-11152-6)
Supplement: Supplementary file 2 — Reporting Summary [file 41467_2019_11152_MOESM2_ESM.pdf]

nature research

Corresponding author(s): Ana Paula Souza and Marco Aurélio Vinolo

Last updated by author(s): May 17, 2019

Reporting Summary

Nature Research wishes to improve the reproducibility of the work that we publish. This form provides structure for consistency and transparency in reporting. For further information on Nature Research policies, see Authors & Referees and the Editorial Policy Checklist.

Statistics

For all statistical analyses, confirm that the following items are present in the figure legend, table legend, main text, or Methods section.

n/a	Confirmed	
☐	☒	The exact sample size (*n*) for each experimental group/condition, given as a discrete number and unit of measurement
☐	☒	A statement on whether measurements were taken from distinct samples or whether the same sample was measured repeatedly
☐	☒	The statistical test(s) used AND whether they are one- or two-sided<br>*Only common tests should be described solely by name; describe more complex techniques in the Methods section.*
☒	☐	A description of all covariates tested
☐	☒	A description of any assumptions or corrections, such as tests of normality and adjustment for multiple comparisons
☐	☒	A full description of the statistical parameters including central tendency (e.g. means) or other basic estimates (e.g. regression coefficient) AND variation (e.g. standard deviation) or associated estimates of uncertainty (e.g. confidence intervals)
☐	☒	For null hypothesis testing, the test statistic (e.g. *F*, *t*, *r*) with confidence intervals, effect sizes, degrees of freedom and *P* value noted<br>*Give P values as exact values whenever suitable.*
☒	☐	For Bayesian analysis, information on the choice of priors and Markov chain Monte Carlo settings
☒	☐	For hierarchical and complex designs, identification of the appropriate level for tests and full reporting of outcomes
☒	☐	Estimates of effect sizes (e.g. Cohen's *d*, Pearson's *r*), indicating how they were calculated

Our web collection on statistics for biologists contains articles on many of the points above.

Software and code

Policy information about availability of computer code

Data collection	Flow cytometry data were collected using BD FACSDiva™ software<br>Real-time PCR data were made using StepOne™ Software v2.3
Data analysis	Flow cytometry data analysis was performed using FlowJo software version 9.7.5 (Tree Star) for Windows<br>Statistical analysis between groups was performed with Graph pad prism version 6<br>Microscopy images were analysed using ImageJ 1.x (NIH)

For manuscripts utilizing custom algorithms or software that are central to the research but not yet described in published literature, software must be made available to editors/reviewers. We strongly encourage code deposition in a community repository (e.g. GitHub). See the Nature Research guidelines for submitting code & software for further information.

Data

Policy information about availability of data

All manuscripts must include a data availability statement. This statement should provide the following information, where applicable:

- Accession codes, unique identifiers, or web links for publicly available datasets
- A list of figures that have associated raw data
- A description of any restrictions on data availability

The source data underlying Figs. 1b, c, d, g, h, j; 2b, c, d, f; 3b-d; 4b, c, d, g, i, j; 5a-d; 6a, b, c, e, f, g, h, I, k, m; 7a, b, c, d, f, h, i, j; 8b-d; 9a, b, c, f, g, i; and Supplementary Fig. 5a, d, e, f are provided in the Source Data file. All other relevant data are available from corresponding authors upon reasonable request.
The script used to run 16S analysis at the CMMR is available on GitHub: CMMR-16S rbiom https://github.com/cmmr/rbiom

Field-specific reporting

Please select the one below that is the best fit for your research. If you are not sure, read the appropriate sections before making your selection.

☒ Life sciences ☐ Behavioural & social sciences ☐ Ecological, evolutionary & environmental sciences

For a reference copy of the document with all sections, see nature.com/documents/nr-reporting-summary-flat.pdf

Life sciences study design

All studies must disclose on these points even when the disclosure is negative.

Sample size	Sample size was estimated to be adequate based on the magnitude and consistency of measurable differences between groups. No calculations were made.
Data exclusions	Conventionally, the data were not excluded. Exclusion occurred only if there was severe abnormality in the data generated and had been identified and confirmed as outliar.
Replication	The number of times each experiment was performed and which statistical analysis was used is indicated in the figure legends.
Randomization	Mice were age matched for each experiment.
Blinding	Investigators were not blinded to mouse infection or treatment. The blinding was made during data analyisis which samples were coded and/or sent to different laboratories to be analysed.

Reporting for specific materials, systems and methods

We require information from authors about some types of materials, experimental systems and methods used in many studies. Here, indicate whether each material, system or method listed is relevant to your study. If you are not sure if a list item applies to your research, read the appropriate section before selecting a response.

Materials & experimental systems

n/a	Involved in the study	
☐	☒	Antibodies
☐	☒	Eukaryotic cell lines
☒	☐	Palaeontology
☐	☒	Animals and other organisms
☐	☒	Human research participants
☒	☐	Clinical data

Methods

n/a	Involved in the study	
☒	☐	ChIP-seq
☐	☒	Flow cytometry
☒	☐	MRI-based neuroimaging

Antibodies

Antibodies used	Anti-CD11c (#561241, clone HL3BD, Biosciences®);<br>Anti-I-Ad/I-Ed (#558593, clone 2G9BD, Biosciences®);<br>Anti-CD86 (#553691, clone GL1, BD Biosciences®);<br>Anti-CD8 (#561092, clone 53-6.7, BD Biosciences®);<br>Anti-CD4 (#553046, clone RM4-5, BD Biosciences®);<br>Anti-CD25 (#552880, PC61, BD Biosciences®);<br>Anti-CD62L (#553152, clone MEL-14, BD Biosciences®);<br>Anti-IL-4 (#562045, clone 11B11, BD Biosciences®);<br>Anti-FoxP3 (#72-5775, clone FJK-16s, eBioscience);<br>Anti-IFNγ (#RM9001, clone XMG1.2, eBioscience);<br>Anti-IL-17a (#506939, clone TC11-18H10.1, BioLegend);<br>Anti-CD45 (#553080, clone 30-F11, BD Biosciences®);<br>Anti-CD326 (EpCAM) (#25-5791-80, clone G8.8, eBioscience);<br>Anti-NFκB p65 (F-6) (#sc-8008, Santa Cruz Biotechnology);<br>Anti-β-actin (#A2228, Sigma Aldrich);<br>Anti-PCNA (#NCL-L-PCNA, Novocastra);<br>Anti-IgG (H+L)-HRP (#61-6520, ThermoFisher Scientific).
Validation	All the monoclonal antibodies listed above are standard reagents used in the field and validated in the literature as cited on the manufacturers websites, as well as by the manufacturers data sheets themselves.

October 2018

Eukaryotic cell lines

Policy information about cell lines

Cell line source(s)	- Human adenocarcinoma alveolar epithelial cells (A549) - obtained from ATCC (CCL-185);<br>- Human embryonic lung fibroblasts (MRC-5) - obtained from ATCC (CCL-171);<br>- Epithelial cells extracted from kidney of African green monkey (Vero) - obtained from ATCC (CCL-81).
Authentication	All the authentication were provided by ATCC.
Mycoplasma contamination	All cell lines were tested for mycoplasma contamination and resulted in negative prior to initiate the experiments.
Commonly misidentified lines<br>(See ICLAC register)	No misidentified cell lines were used.

Animals and other organisms

Policy information about studies involving animals; ARRIVE guidelines recommended for reporting animal research

Laboratory animals	In this was used:<br>- Female BALB/c;<br>- Male and female type 1 interferon receptor deficient (Ifnar-/-) 129/Sv;<br>- Male and female wild type 129/Sv;<br>- Female Rag-1 deficient (Rag1-/-);<br>- Female GPR43-deficient (Gpr43-/-) C57BL/6;<br>- Female C57BL/6.<br><br>*All at age 6-8 weeks.
Wild animals	Wild animals were not used in the study.
Field-collected samples	This study did not involve samples collected from the field.
Ethics oversight	All animal procedures were performed in accordance with protocols approved by Animals Ethics Committee of UNICAMP CEUA/ UNICAMP (protocols 4022-1 and 4599-1).

Note that full information on the approval of the study protocol must also be provided in the manuscript.

Human research participants

Policy information about studies involving human research participants

Population characteristics	Healthy full-term infants (< 1 year of age) presenting with bronchiolitis. Diagnosis of RSV infection was performed by trained pediatricians based on clinical outcomes (oxygen saturation, Th2 polarization, RSV titer and pCO2).
Recruitment	Only RSV positive subjects (246 severe and 172 mild) were included for the study. Previously healthy full-term infants with bronchiolitis were recruited to the study if their oxygen saturation upon enrollment was lower than 93% when breathing room air. Exclusion criteria included known or suspected impairment of immunological function, major congenital oral malformations, chronic lung disease, cardiac disease, prematurity (gestational age of less than 37 weeks), neuromuscular disorders affecting swallowing, and known or suspected coagulation disorders or bleeding tendency.
Ethics oversight	The Institutional Review Boards of participating hospitals in Buenos Aires and Johns Hopkins University approved the protocol and the study conformed to standards indicated by the Declaration of Helsinki. Informed consent was obtained from the parents of each enrolled infant.

Note that full information on the approval of the study protocol must also be provided in the manuscript.

Flow Cytometry

Plots

Confirm that:

☒ The axis labels state the marker and fluorochrome used (e.g. CD4-FITC).

☒ The axis scales are clearly visible. Include numbers along axes only for bottom left plot of group (a 'group' is an analysis of identical markers).

☒ All plots are contour plots with outliers or pseudocolor plots.

☒ A numerical value for number of cells or percentage (with statistics) is provided.

Methodology

Sample preparation	The sample preparation is better described in the Methods section. Briefly, isolated cells from lung or axillary lymph nodes were incubated with Mouse Fc Block (#553141 BD Biosciences®) for 20 min and then stained with surface antibodies. Then, for intracellular staining, cells were fixed with cytofix/cytoperm (BD Biosciences®) and stained with intracellular antibodies. For cell sorting samples were only surface stained and were not fixed. Erythrocytes were lysed using a hypotonic solution.
Instrument	Samples were analyzed on Gallios (Beckman Coulter®), BD FACS Verse (BD Biosciences®) and BD FACSAria (BD Biosciences®).
Software	FlowJo software version 9.7.5 (Tree Star) for Windows.
Cell population abundance	All cell population abundances are indicated in the figure plots.
Gating strategy	The initial gate strategie started capturing all cells by FSC vs SSC area, single cells by FSC height versus area, followed by a second single cells through SSC width vs SSC area. Gating strategies differed by experiment. Figures exemplifying the gating strategy are provided in the Supplementary Iformation.

☒ Tick this box to confirm that a figure exemplifying the gating strategy is provided in the Supplementary Information.
